# Modifications of Porphyrins and Hydroporphyrins for Their Solubilization in Aqueous Media

**DOI:** 10.3390/molecules22060980

**Published:** 2017-06-13

**Authors:** Michael Luciano, Christian Brückner

**Affiliations:** Department of Chemistry, University of Connecticut, Storrs, CT 06269-3060, USA; michael.luciano@uconn.edu

**Keywords:** water-solubility, porphyrin, chlorin, bacteriochlorin, PEGylation, bioconjugation, photodynamic therapy, bioimaging

## Abstract

The increasing popularity of porphyrins and hydroporphyrins for use in a variety of biomedical (photodynamic therapy, fluorescence tagging and imaging, photoacoustic imaging) and technical (chemosensing, catalysis, light harvesting) applications is also associated with the growing number of methodologies that enable their solubilization in aqueous media. Natively, the vast majority of synthetic porphyrinic compounds are not water-soluble. Moreover, any water-solubility imposes several restrictions on the synthetic chemist on when to install solubilizing groups in the synthetic sequence, and how to isolate and purify these compounds. This review summarizes the chemical modifications to render synthetic porphyrins water-soluble, with a focus on the work disclosed since 2000. Where available, practical data such as solubility, indicators for the degree of aggregation, and special notes for the practitioner are listed. We hope that this review will guide synthetic chemists through the many strategies known to make porphyrins and hydroporphyrins water soluble.

## 1. Introduction

The utility of porphyrins and hydroporphyrins (chlorins and bacteriochlorins) in a variety of biomedical applications has been demonstrated [1]. These include photodynamic cancer therapy [2,3,4,5], antifungal photodynamic therapy [6], their use as photoantimicrobials [7], as fluorescence and photo-acoustic imaging agents [5,8,9,10,11,12,13,14], (single) cell imaging [15] and fluorescence labelling [16], and as in vivo chemosensors [17,18]. In addition, they have shown value in a number of technical applications, such as chemosensing [17], electronic devices and materials [19,20,21], catalysis [22], photocatalysis [23,24], and light harvesting [25,26,27,28]. These applications rest on the favorable optical, chemical and electronic properties of these porphyrinoids: their intense absorbance in the visible to near infrared (NIR) region, high fluorescence or singlet oxygen quantum yields, good photoacoustic signal generation efficiency, ease of ejection of a photo-excited electron, etc. Many of these applications would greatly benefit from an inherent solubility of the porphyrins in aqueous media, i.e., not requiring any surfactants or liposome vesicles to mediate solubility. However, even though the methodologies for preparing synthetic porphyrins [29,30], hydroporphyrins [2,31,32,33,34], and their analogues [34,35,36,37,38,39], have advanced enormously in the past decades, the vast majority of the synthetic porphyrinoids presented are not natively water-soluble, preventing—or at least complicating—their utilization in, for example, biological contexts.

Initial efforts to impart water-solubility relied on the utilization of *meso*-pyridyl and *meso*-*p*-sulfonatophenyl-substituents in (mostly) symmetric porphyrins. These porphyrins were based on Adler syntheses of tetrapyridyl- and tetraphenylporphyrin, followed by quarternization with a suitable alkyl halide or direct sulfonation, respectively. Albeit simple, both methods have drawbacks. They are difficult to translate to β-alkyl-substituted porphyrins, the sulfonation reaction conditions are harsh and therefore incompatible with many hydroporphyrin chromophores or substituents that convey desirable properties onto the porphyrin. This led to the development of a range of alternative methods to impart water-solubility that are mild, selective, and that generate the water-soluble porphyrin derivative late in the synthetic sequence, thus facilitating its isolation and purification.

Rather than attempting to be comprehensive, we aim to present here a tutorial-style review that highlights recent (since ~2000) synthetic strategies. Where data are available, we compare the resulting solubilities, and report on other notable practical considerations. This summary extends a review on water-soluble porphyrins published in the year 2000 [40] that provides a detailed account of the acid-base properties and aggregation behavior of conventional water-soluble porphyrins. A 2014 review on amphiphilic porphyrins by Gryko and co-workers [41] is also available; overlap with this review is minimized and these reviews are thus complementary. Porphyrins linked to sugars, many of which are also water-soluble, have been reported [41,42,43]. We chose not to include them here because glycosylation strategies are frequently not driven by achieving water-solubility but to increase tumor targeting; they were also reviewed in the context of amphiphilic porphyrins [41]. Water-soluble porphyrins conjugated to biomolecules (e.g., oligonucleotides and peptides), appended to functionalities such as cyclodextrans [44], crown ethers [45,46], porphyrin nanoparticles [47,48,49,50], or porphyrins that were solubilized with the help of surfactants or vesicles [51,52,53] are also beyond the scope of this discussion. Likewise, we do not cover the solubilization of expanded porphyrins or most other porphyrinoids.

**Scheme 1:**
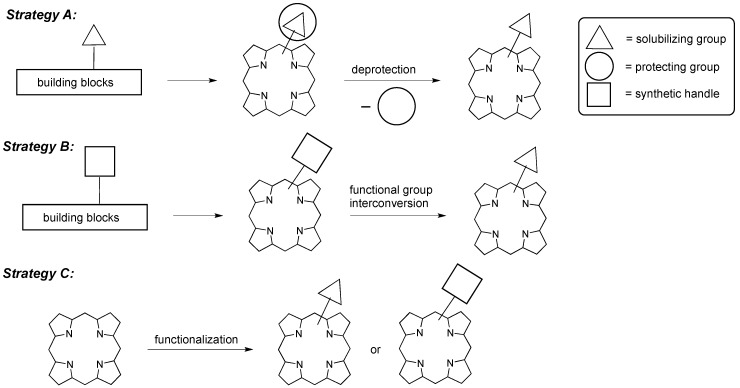
Principle strategies toward hydrophilic porphyrin derivatives.

The review is organized along the charge the solubilization groups introduce: Cationic (amine/ammonium, pyridyl/pyridinium), anionic (sulfonate, phosphonate, carboxylic acid/carboxylate) and neutral (polyethylene glycol (PEG) chains). Each section discusses the examples loosely in increasing order of complexity and the position of the solubilizing group. Three principle strategies for the introduction of solubilizing groups can be distinguished (Scheme 1). *Strategy A* encompasses the synthesis of a porphyrin macrocycle using building blocks bearing solubilizing groups (protected or not). *Strategy B* involves the synthesis of a porphyrin macrocycle with precursor functionalities, that can later be manipulated to hydrophilic groups. *Strategy C* employs synthetic modifications of an already-formed porphyrin to impart water solubility.

## 2. Water-Soluble Porphyrins Bearing Cationic Substituents

Cationic porphyrins were among the first synthetic water-soluble porphyrins to be discovered [54]. These early methods of solubilization relied on the *N*-alkylation of *meso*-tetrapyridylporphyrins and their metal complexes (see also Section 2.2.1) [41]. These types of cationic porphyrins were widely studied for their association with the polyanion DNA [55,56], as part of the quest for new photosensitizers for photodynamic therapy (PDT), and for their promise as photoantimicrobials for gram positive and gram negative bacteria [57]. Tetra-pyridyl/pyridiniumporphyrins are also commercially available [58].

### 2.1. Amine/Ammonium Functionalized Porphyrins

One possibility for the preparation of cationic water-soluble porphyrins and hydroporphyrins is through derivatization with amines or polyamines. Porphyrinoids with amine functional groups may be soluble in aqueous media at low pH, but quarternization of the amines with suitable electrophiles is frequently employed to furnish freely water-soluble derivatives. The resulting (poly)cationic derivatives are usually purified by crystallization protocols or reverse phase chromatography. Ion-exchange chromatography offers an option to subsequently exchange the halide counter ion (typically Br^−^ or I^−^) to another anion [59].

A number of symmetric and asymmetric amphiphilic *meso*-aryl porphyrins bearing amine functional groups have been reported [41]. The strategies toward their generation often rely on Adler or Lindsey porphyrin syntheses incorporating amine-containing building blocks, or through modification of other functionalities, such as phenols.

**Scheme 2:**
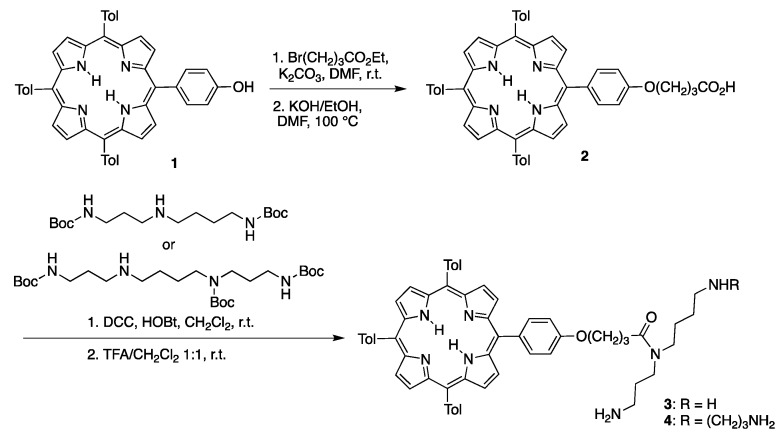
Spermine- and spermidine-porphyrin conjugates, as described by the group of Krausz [60]. DCC = dicyclohexylcarbodiimide, HOBt = 1-hydroxybenzotriazole.

Krausz and co-workers, for example, synthesized polyamine-*meso*-arylporphyrin conjugates by DCC-mediated amide-coupling of carboxylic acid-functionalized A_3_B porphyrin **2**, made by functionalization of phenol **1** with di-Boc-protected-spermidine or tri-Boc-protected-spermine (Scheme 2) [60]. The ultimate starting A_3_B porphyrin **1** was synthesized using a mixed aldehyde Adler condensation, followed by alkylation of the phenolic oxygen with ethyl 4-bromobutanoate and saponification. The thus generated polyamines were submitted to standard Boc-deprotection conditions to afford the polyamine-porphyrin conjugates **3** and **4**, respectively.

**Scheme 3:**
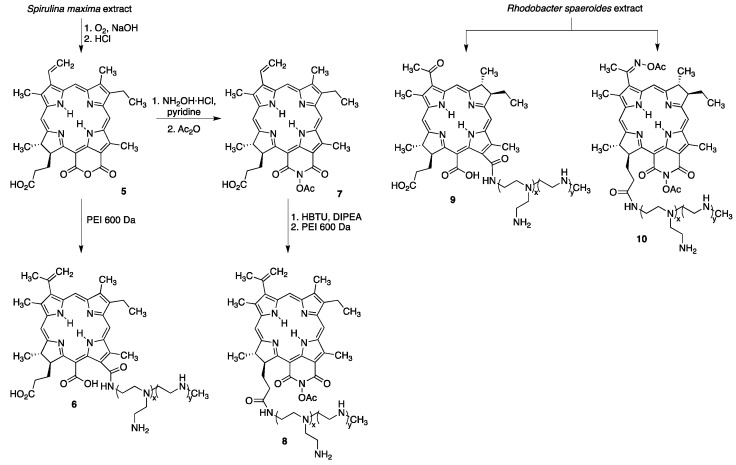
Chlorin and bacteriochlorin polyamines derived from *Spirulina maxima* and *Rhodobacter spaeroides*, respectively, as described by the group of Sol [61]. PEI = polyethyleneimine (PEI, MW 600 Da), HBTU = (2-(1*H*-benzotriazol-1-yl)-1,1,3,3-tetramethyluronium hexafluorophosphate), DIPEA = *N*,*N*-diisopropylethylamine.

Bacteriochlorins derived from natural sources and bearing an anhydride functionality could also be derivatized by amide formation with polyamines [61]. Sol and co-workers prepared water-soluble chlorin polyamine derivative **6** through amidation of a chlorin extracted from *Spirulina maxima* (Scheme 3). The polyamine groups were introduced by nucleophilic ring opening of anhydride **5** with polyethyleneimine (PEI, MW 600 Da). Alternatively, polyamination was performed at the propionic acid side chain. The anhydride moiety in chlorin **5** was first converted to an acetate-protected cycloimide chlorin **7** by treatment with hydroxylamine, followed by acylation. The subsequent polyamination at the propionic acid side chain required activation of the carboxylic acid moiety, followed by reaction with PEI. In a similar fashion, water-soluble polyaminated bacteriochlorins **9** and **10** were prepared starting from bacteriochlorins extracted from *Rhodobacter spaeroides*. In water, the resulting compounds exhibited split and/or broadened Soret bands, indicating some degree of aggregation.

**Scheme 4:**
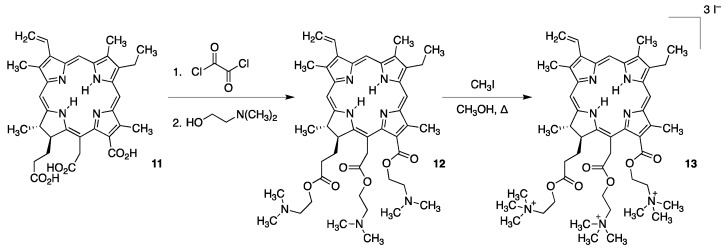
Cationic esters of chlorin e6 with terminal ammonium groups, as described by the group of Inoue [62].

Amine groups were also introduced to porphyrins bearing carboxylic acid groups by esterification with an amino-alcohol. An example of this strategy was the preparation of a tricationic water-soluble ester of chlorin e6 **11** (Scheme 4) [62]. Methyl pheophorbide *a* was saponified, and the acid groups in product **11** were activated as acid chlorides and esterified with *N*,*N*-dimethylamino-ethanol to provide triamine **12**. Quarternization by reaction with CH_3_I gave the tricationic water-soluble chlorin derivative **13** [62].

**Scheme 5:**
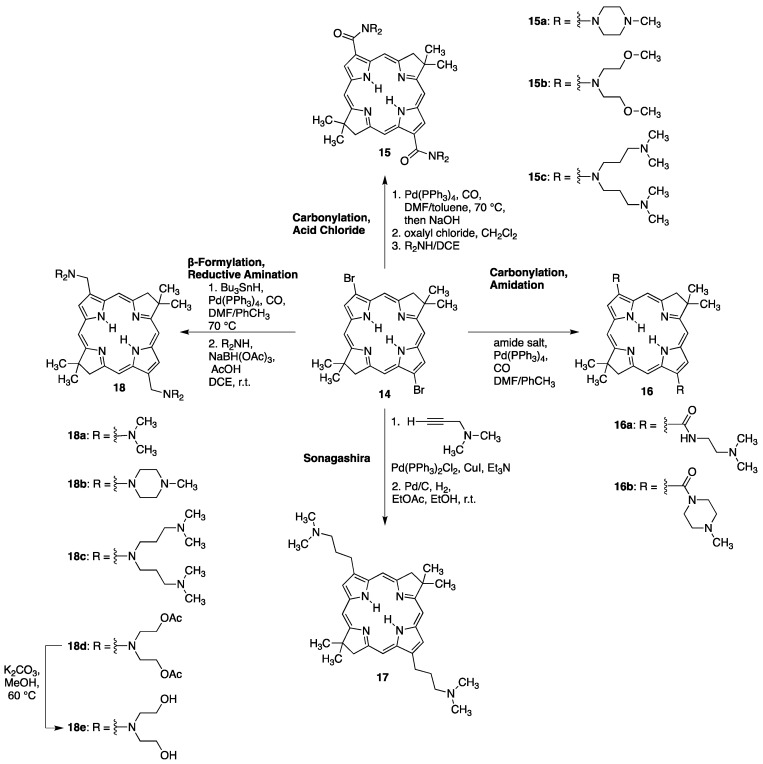
β-substituted cationic ammonium bacteriochlorin derivatives, as described by the group of Lindsey [63]. DCE = dichloroethane.

Amine groups have been introduced to the β-positions of hydroporphyrins by transition metal-mediated coupling strategies using β-bromo precursors. Thus, Lindsey and co-workers prepared a range of water-soluble cationic bacteriochlorin derivatives utilizing the versatile 3,13-dibromo-bacteriochlorin building block **14**. The bromo groups are susceptible to a variety of transition metal-mediated C-C bond forming coupling reactions (Scheme 5) [63]. For example, bacteriochlorins **15a**–**15c** were prepared from **14** through a carbonylation → acid chloride reaction with secondary amines. Additionally, reaction of amide salts with **14** in a carbonylation → amidation sequence gave the aminobacteriochlorins **16a** and **16b**. Sonogashira coupling of **14** with an ethynyl-amine, followed by reduction gave the bacteriochlorin **17** with amine-terminated alkyl chains. Lastly, a variety of secondary amines were employed in a β-formylation → reductive-amination strategy to produce amine-functionalized bacteriochlorins **18a**–**18e**. The resulting amines were amenable to purification by silica gel column chromatography (EtOAc or CH_2_Cl_2_/MeOH/NH_4_OH as eluents). Subsequent *N*-alkylation of the amines was achieved using standard conditions, and the water-soluble cationic products that precipitated from the CHCl_3_ reaction mixtures were isolated by precipitation and centrifugation. Some of these compounds were investigated for their efficacy as antimicrobial photosensitizers [64].

**Scheme 6:**
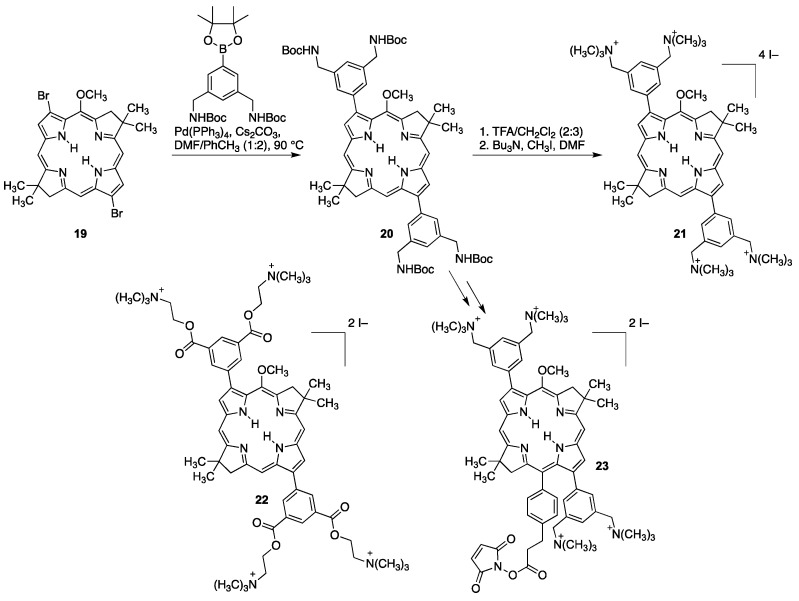
β-Substituted cationic ammonium bacteriochlorin derivatives prepared by Suzuki coupling, as described by the group of Lindsey [65].

Lindsey and co-workers also used similar dibromobacteriochlorin **19** to directly introduce arylamines to the β-positions via Suzuki couplings (Scheme 6) [65]. After the coupling of dibromo-bacteriochlorin **19** with an aryl boronic ester bearing two Boc-protected amines, the Boc groups in product **20** were removed, and the free amine quarternized by treatment with CH_3_I to afford the final ammonium-functionalized bacteriochlorin product **21**.

Related compounds with terminal amine functional groups, such as 3,13-diaryl bacteriochlorin **22**, were prepared using similar Suzuki coupling strategies. Bioconjugatable derivatives of these compounds carrying an NHS-ester functional group, such as **23**, were also prepared by a regio-selective *meso*-bromination → Suzuki coupling sequence (see also Section 2.2.2) [65].

The group of Lindsey also prepared a range of amphiphilic bacteriochlorins of limited aqueous solubility bearing one amine side chain by reductive amination of a β-formylated bacteriochlorin (see also Scheme 5) [66]. A representative example is bacteriochlorin **24**, which possesses an ammonium-terminated dialkyl chain, a so-called swallowtail functionality. This moiety was designed to project solubilizing groups above and below the plane of the macrocycle, leading to facial encumbrance that inhibits macrocycle stacking and increases hydrophilicity. A similar strategy was used to solubilize *meso*-aryl porphyrins in organic solvents [67,68].

**Figure 1:**
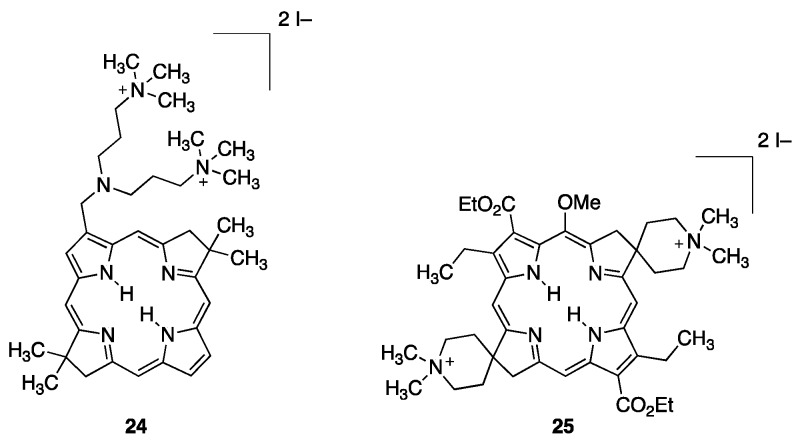
Molecular structures of ammonium-substituted bacteriochlorins **24** and **25**.

Other ammonium-based water-soluble derivatives were also prepared by the Lindsey group by introduction of spiro-linked piperidine moieties at the β-positions of a bacteriochlorin [69]. The integral spiropiperidine functional groups were quarternized using standard conditions to furnish water-soluble derivatives, such as bacteriochlorin **25** (Figure 1).

**Scheme 7:**
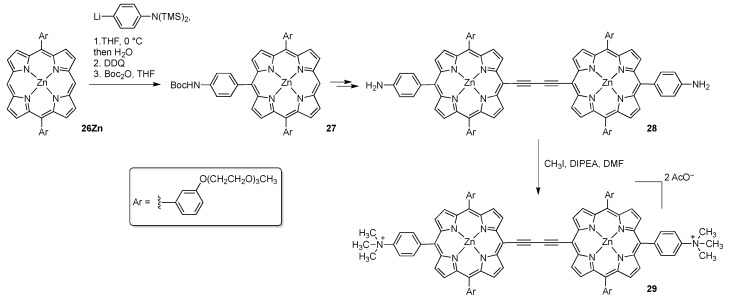
*meso*-Aryl-ammonium bis-acetylene linked porphyrin dimers prepared via Senge arylation, as described by the Anderson group [70]. DIPEA = *N*,*N*-diisopropylethylamine.

The direct introduction of ammonium groups using a nucleophilic Senge arylation strategy was performed by Anderson and co-workers on 1,15 diaryl porphyrin **26Zn** (Scheme 7) [70]. Porphyrin **26** was functionalized at the *meso*-position with a Boc-protected amino-phenyl group. The resulting derivative **27** was carried through multiple steps to afford porphyrin dimer **28** that was rendered water-soluble by quarternization of the amine functionality with CH_3_I.

### 2.2. Porphyrins Carrying Pyridyl/Pyridinium Groups

#### 2.2.1. *meso*-Tetrakispyridiniumporphyrins

**Scheme 8:**
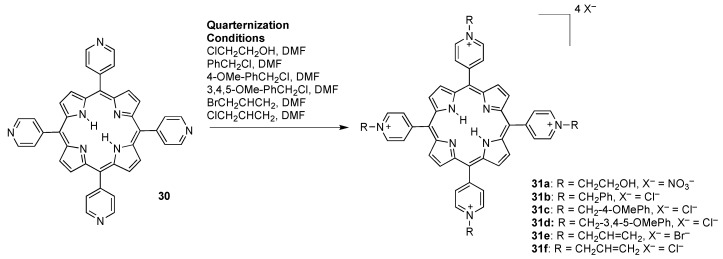
Quarternization options of *meso*-tetrakis(4-pyridyl)porphyrin.

*meso*-Tetrapyridylporphyrins and the corresponding *meso*-tetrakis(methylpyridinium)-porphyrins are long known, and their intercalation into DNA was studied in detail [55,56]. Pyridinium porphyrins continue to attract attention as next generation photosensitizers for PDT. Various new *meso*-tetrakispyridiniumporphyrin derivatives were prepared using a range of electrophiles to quarternize the pyridine nitrogens (Scheme 8).

Orlandi and co-workers prepared *meso*-tetrakis(4-pyridinium)porphyrins (**31**) by reaction of *meso*-tetra(4-pyridyl)porphyrin (**30**) with various benzyl chlorides to study the effect of polar alkylating groups on their antibacterial activity [71]. The benzylation conditions required were more vigorous (reflux in DMF for 24 h) than typical for pyridine alkylations using alkyl halides. The water-soluble products were recovered by precipitation with Et_2_O and filtration. Similarly, Ghazaryan and co-workers prepared a variety of *meso*-substituted *N*-substituted tetrapyridiniumporphyrins with different central metals (Ag, Zn, Co and Fe) and alkylating agents (allyl-, oxyethyl-, butyl- and methallyl-substituted derivatives) [72]. A PEGylated derivative of *meso*-tetrakispyridiniumporphyrin was also prepared by alkylation of *meso*-tetra(4-pyridyl)-porphyrin with a short PEG-bromide [73,74].

**Figure 2:**
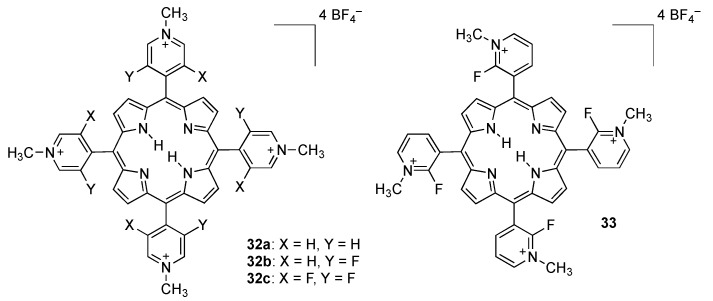
Molecular structures of *meso*-pyridinium-substituted porphyrins **32** and **33**.

Fluorinated derivatives of *m*- or *p*-linked *meso*-pyridyl porphyrins were prepared along Adler synthesis routes [75]. *N*-Alkylation of the pyridyl substituents by treatment with (CH_3_)_3_O^+^BF_4_^−^ afforded the tetra-alkylated products **32** and **33**, respectively, as their tetrafluoroborate salts (Figure 2).

**Scheme 9:**
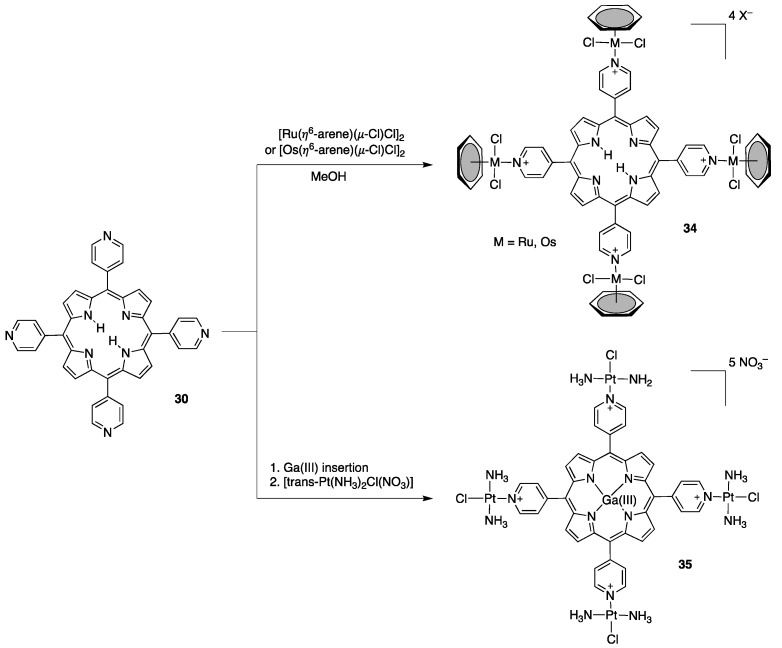
M-Arene complexes of *meso*-tetra(4-pyridyl)porphyrin, as described by the groups of Therrien [76,77], and Odani [78].

In search of dual-action photo- and cytotoxic anti-cancer agents, various metal complexes were coordinated to the outer pyridyl nitrogens in tetrapyridylporphyrins (Scheme 9). Ruthenium, rhodium and iridium complexes were thus prepared by Therrien and co-workers [76] by treatment of *meso*-tetrapyridylporphyrin **30** with various metal arene salts to afford the water-soluble organic metallic complexes of type **34**. They also prepared mixed arene-ruthenium substituted porphyrins in a similar manner, producing mono- and tetrametallated derivatives of 3- and 4-tetrapyridyl-porphyrins [77]. Both *cis*- and *trans*-platin conjugates of porphyrins were prepared by treatment of **30** with *cis*- or *trans*-Pt(II) complexes, respectively [77]. The gallium complexes of these compounds (such as **35**) were prepared by Odani and co-workers [78] and also tested as photosensitizers for cancer therapy. In addition, tetrapyridylporphyrins with ruthenium nitrosyl groups substituted at their periphery were reported by the group of Alessio, generated by reaction of **30** with ruthenium nitrosyls and purified by column chromatography (CHCl_3_/5% EtOH as eluent) [79].

#### 2.2.2. A_3_B and Other *meso*-Pyridiniumporphyrins

**Scheme 10:**
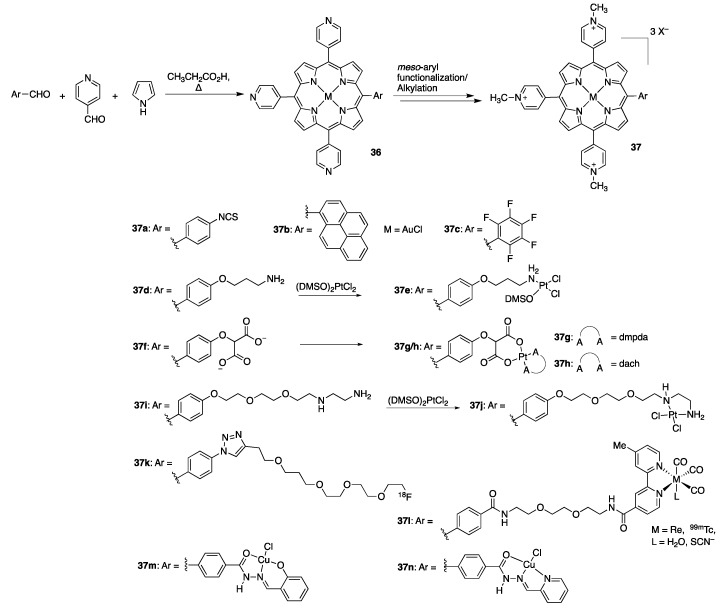
A_3_B tri-pyridyl porphyrins prepared by Adler synthesis and subsequent synthetic modification. dmpda = *N*,*N′*-dimethyl-1,3-propanediamine, dach = 1,2-diaminocyclohexane.

A number of symmetrically substituted pyridylporphyrins have been prepared in the search for the next generation of anticancer PDT agents. However, to further optimize their tumor-targeting and biodistribution properties, the porphyrin needs to be amenable to further modifications that will allow, for example, the conjugation of the porphyrin to a targeting molecule. Thus, a variety of examples emerged that use A_3_B porphyrins bearing three *meso*-pyridinium groups as the water-soluble framework of the chromophore, with the fourth *meso*-aryl ring available for further manipulations (Scheme 10). The parent A_3_B porphyrins are typically synthesized as statistical mixtures by Adler condensation of pyridylaldehyde, the desired arylaldehydes, and pyrrole, followed by chromatographic separation of the product mixture. The A_3_B porphyrin precursors are functionalized at the remaining *meso*-aryl group and *N*-alkylated with CH_3_I, with the order of these operations depending on the functionality present (see below).

Using this strategy, a pyrene-substituted trispyridyl A_3_B porphyrin could be prepared [80] (Scheme 10). Its *N*-alkylation with methyl *p*-toluenesulfonate in refluxing CHCl_3_/CH_3_NO_2_ afforded the tris-*N*-methyl pyridiniumporphyrin **37b**. The group of Boyle synthesized tripyridyl A_3_B porphyrin **37a** with the fourth aryl ring bearing a bioconjugatable isocyanate group.

The *N*-alkylation step (standard CH_3_I alkylation conditions) needed to be performed after the formation of the isocyanate group to avoid unwanted *N*-alkylation of the amine precursors [81]. A number of *p*-substituted phenyl derivatives allowed a wide range of functionalizations. For instance, Song et al. synthesized A_3_B pyridyl porphyrin **37j** with the fourth aryl group bearing a Pt(II) complex linked to the hydroxyl group of the *meso*-phenol group by a triethylene glycol linker [82]. *N*-Alkylation of the three pyridyl groups, followed by Boc-deprotection and complexation with the Pt(II) precursor, yielded the A_3_B platinum-porphyrin conjugate **37j**. A similar approach was taken by the group of Alessio to synthesize water-soluble ^99m^Tc(I)/Re(I)-porphyrin conjugates, such as **37l**, utilizing an amide-linked spacer between the aryl group and the ^99m^Tc(I)/Re(I) ion [83,84]. The water-soluble derivatives were purified by normal phase silica gel column chromatography (CH_2_Cl_2_/MeOH mixtures). These types of compounds can also be conjugated to cancer-targeting peptides for use in cancer therapy [85]. Cu(II)-based acylhydrazone porphyrin-derivatives **37m** and **37n** could be obtained using a similar strategy [86]. The group of Boyle synthesized an A_3_B porphyrin that could be further functionalized to establish an azide group [87]. The azide functionality was used in a click reaction to introduce an ^18^F-terminated short PEG chain to generate water-soluble porphyrin **37k** for PDT/PET theranostic applications [87]. After *N*-methylation of the pyridyl nitrogens, the subsequent synthetic steps required precipitation of the product using Et_2_O/MeOH mixtures. The final products were purified by passing through a column of neutral alumina.

**Figure 3:**
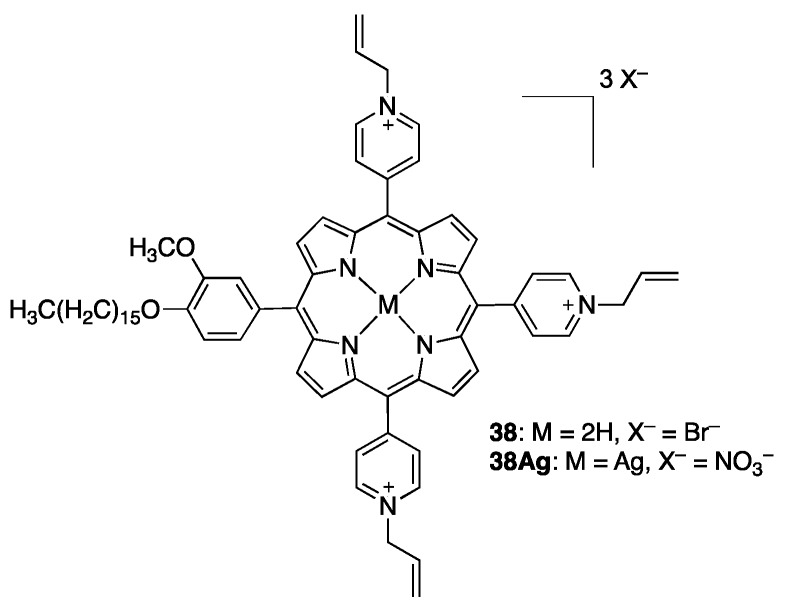
Molecular structure of *meso*-pyridinium-substituted porphyrin **38**/**38Ag**.

Gasparyan and co-workers synthesized an A_3_B amphiphilic trispyridylporphyrin with one *meso*-aryl group carrying a long alkyl chain using a mixed aldehyde Adler condensation [88]. Quarternization of the pyridyl groups with 3-bromopropene provided the water-soluble vinyl-substituted trispyridiniumporphyrins **38** and **38Ag** (Figure 3) that were isolated by precipitation with acetone.

**Scheme 11:**
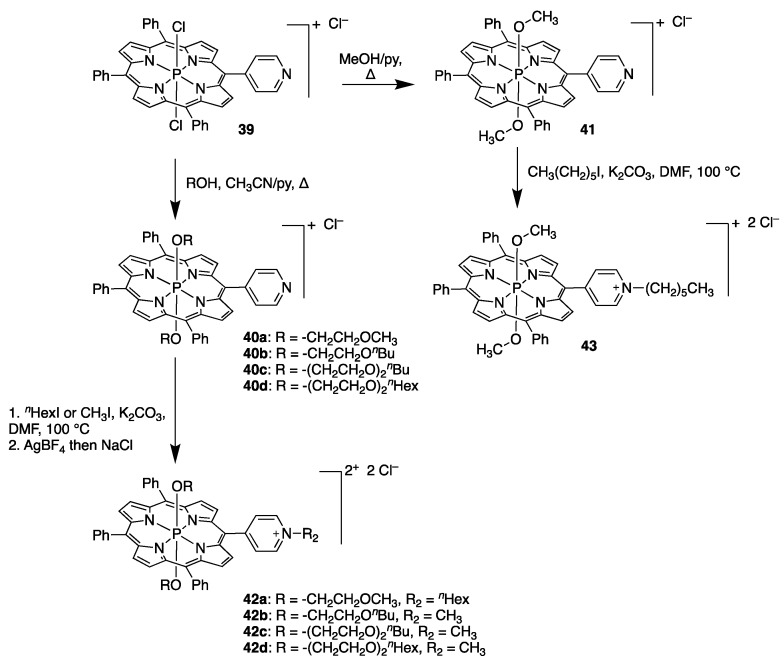
Water-soluble AB_3_ phosphorus porphyrins, as described by the group of Yasuda [89].

Yasuda and co-workers prepared AB_3_ phosphorus porphyrin **39** [89]. Its axial chloro-ligands could be exchanged for simple alcohols and oligoethylene glycol chains to generate hydrophilic phosphorus porphyrins **40** and **41**. *N*-Alkylation of the *meso*-pyridyl group in **40a**–**40d** or **41** then furnished water-soluble derivatives, with solubility in water of around ~10 mM for compounds **42a**–**42d** and **43**. (Scheme 11) [89]. An equivalent methodology was used to introduce axial PEG chains [90].

**Scheme 12:**
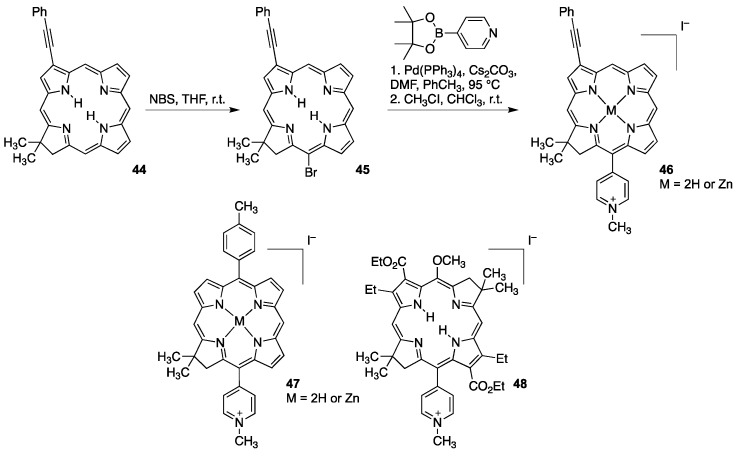
Amphiphilic *meso*-pryridyl chlorins and bacteriochlorins, as described by the group of Lindsey [91].

Using a fundamentally different strategy toward *meso*-pyridyl porphyrins, pyridyl substituents can be introduced to *meso*-brominated porphyrins by using Suzuki couplings. For instance, the group of Lindsey reported the synthesis of a series of amphiphilic hydroporphyrins prepared by Suzuki coupling of a pyridyl boronic ester with *meso*-bromo-chlorins or bacteriochlorins [34]. Key chlorin building block **45** was prepared by regioselective *meso*-bromination of chlorin **44** using NBS (Scheme 12) [91]. In the final step of the synthesis, the pyridyl functional group was quarternized with CH_3_I to furnish bacteriochlorin **46**. This strategy is flexible and tolerant of a number of other substituents on the hydroporphyrin framework. Thus, chlorin **47** and bacteriochlorin **48** were prepared along analogous routes.

**Scheme 13:**
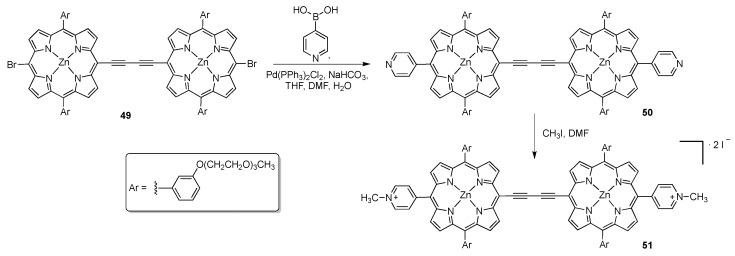
Preparation of a *meso*-pyridinium-functionalized bis-acetylene linked porphyrin dimer, as described by the group of Anderson [70].

In an analogous manner, Anderson and co-workers employed a Suzuki coupling strategy to introduce pyridyl functional groups to *meso*-dibrominated bis-acetylene linked porphyrin dimer **49** (Scheme 13) [70]. After coupling with 4-pyridinylboronic acid, both *meso*-pyridyl functional groups in **50** were methylated under standard conditions to afford the dicationic product **51**. This water-soluble porphyrin dimer was purified by precipitation from DMF/Et_2_O.

Anderson and coworkers also introduced pyridyl substituents to the *meso*-position of *trans*-AB porphyrins by Sonogashira coupling of *meso*-alkynyl porphyrin with 4-iodopyridine. The pyridyl groups were then quarternized with the electrophiles CH_3_I, 1,4-butane sultone or 5-iodo-*N*,*N*,*N*-tri-methylpentan-1-ammonium [92,93,94].

#### 2.2.3. Other Pyridyl-Substituted Porphyrins

**Figure 4:**
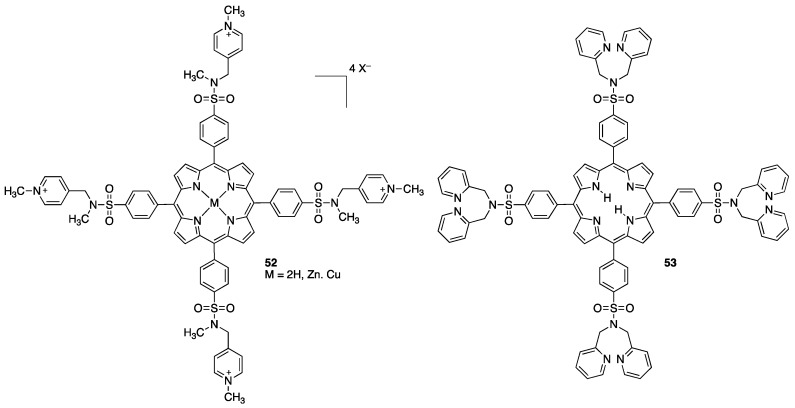
Molecular structures of sulfonamide-linked pyridinium/dipicolylamine-substituted porphyrins **52** and **53**.

Porphyrins bearing alkylated pyridinium substituents in positions not directly linked to the *meso*- or β-positions of the macrocycle were also prepared. For example, Marzilli and co-workers synthesized porphyrins **52** (Figure 4) with the pyridyl groups linked through sulfonamide linkages to the *p*-phenyl positions of *meso*-tetraarylporphyrins [59,95]. They investigated whether distant pyridinium groups are equally competent in mediating DNA-intercalation. Dipicolylamine (DPA) substituents could also be linked through the sulfonamide group (**53**), to prepare rhenium-based radiopharmaceuticals [96].

**Scheme 14:**
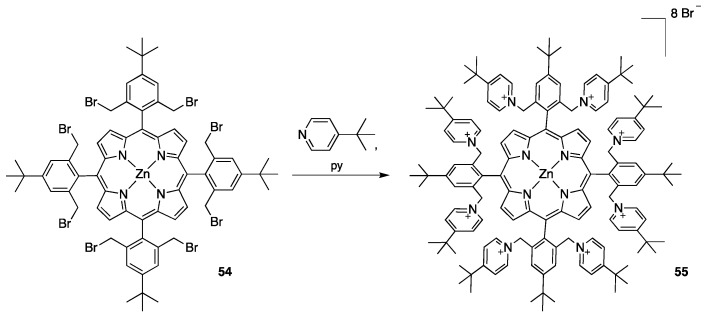
An octapyridiniumporphyrin, as described by the group of Prato [97].

A remarkable octapyridiniumporphyrin **55** was prepared by Prato and co-workers [97] by treatment of *meso*-tetrakis(2,6-bis-(bromomethyl)-4-*t*-butylphenyl) porphyrin **54** with *t*-butylpyridine (Scheme 14). The octapyridyl porphyrin **55** was purified by crystallization from Et_2_O/MeOH. The octacationic compound **55** was used in studies toward assemblies for light-harvesting applications.

**Figure 5:**
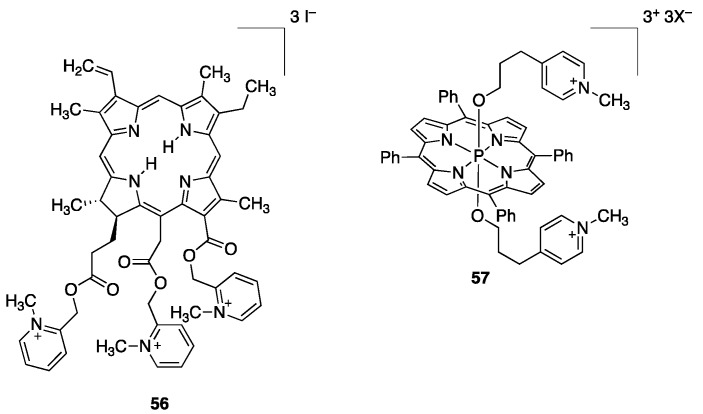
Molecular structures of pyridinium-substituted chlorin **56** and phosphorus porphyrin **57**.

The introduction of pyridinium groups can also be achieved by esterification of a β-alkylester with a suitable pyridine-derivatized alcohol. Thus, cationic water-soluble ester **56** was prepared from chlorin e6 (**11**) by esterification with 2-(2-hydroxyethyl)pyridine, followed by quarternization with CH_3_I (cf. Scheme 4) [62]. Derivatives **56** (Figure 5) and related **13** possess sharp absorbance spectra in Tris-HCl buffer at pH 7.6, indicating little aggregation of the chromophores.

Pyridinium substituents have also been introduced in the axial positions of phosphorus and antimony *meso*-tetraphenyl porphyrins by axial exchange using a pyridine-derivatized alcohol (cf. to Scheme 11) [89,98]. Alkylation of the axial pyridyl moiety with a range of alkyl bromides furnished derivatives such as **57** (Figure 5), with a water-solubility of ~3 mM [89].

#### 2.2.4. *meso*-Imidazolium Porphyrins

**Scheme 15:**
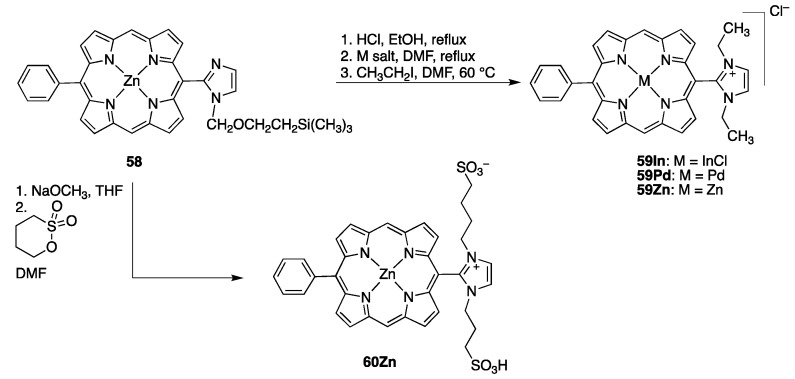
*meso*-Imidazolium porphyrins, as described by the groups of Lindsey and Hamblin [100,101].

The synthesis of porphyrins carrying *N*-alkylated *meso*-imidazolyl functionalities represents another strategy for the preparation of cationic porphyrins [99]. Lindsey and co-workers prepared porphyrins with one or two *meso*-imidazolyl groups by starting from imidazole-functionalized dipyrromethane precursors [100,101]. Mono-imidazolyl porphyrin **58** was alkylated with various alkylating reagents. The resulting imidazolium porphyrins were studied for their use in photodynamic therapy. The zwitterionic imidazolium sulfonate porphyrin **60Zn**, for example, is the product obtained when 1,4-butane sultone was used as the alkylating reagent (Scheme 15) [100].

## 3. Water-Soluble Porphyrins Bearing Anionic Substituents

### 3.1. Porphyrins with Carboxylate Functional Groups

**Scheme 16:**
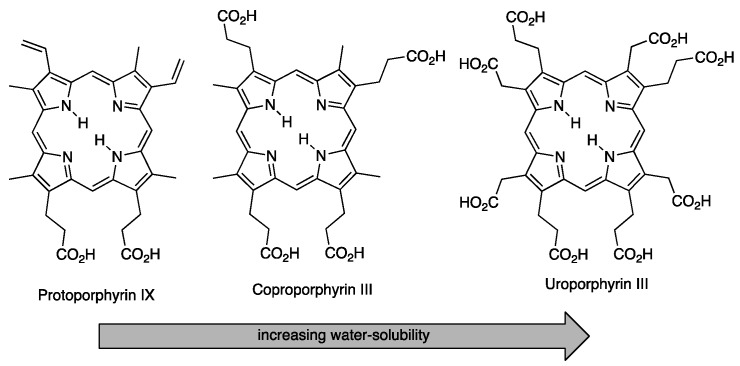
Increasing water-solubility of the naturally occurring porphyrins with increasing number of carboxylic acid functionalities.

Many naturally occurring porphyrins contain acetic acid or propionic acid side chains [102]. With increasing number of acid side chains, their solubility in aqueous solution increases, with the uroporphyrins being freely soluble, particularly in solutions of basic pH (Scheme 16) [102].

This suggests that the introduction of carboxylic acid functional groups to the periphery of porphyrins might be an effective way of furnishing water-soluble derivatives. This is, in fact, also observed. To simplify the purification of the porphyrins, the carboxylate groups are often masked throughout the synthesis as esters and are revealed by saponification in the final step.

**Scheme 17:**
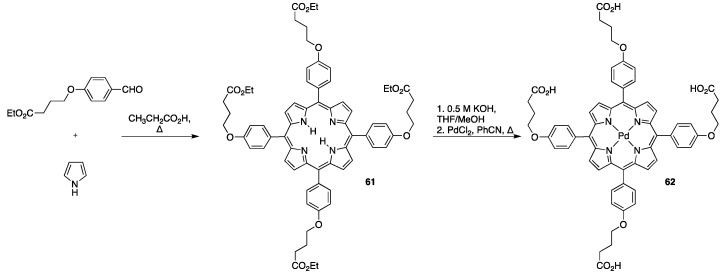
Carboxylated *meso*-aryl porphyrins, as described by the group of Skondra [106].

*meso*-Tetrakis(4-carboxyphenyl)porphyrin, and its ester, are well known, and readily synthe-sized by condensation of the corresponding aldehydes with pyrrole [103,104,105]. They are also commercially available [58]. Carboxylate groups may also be introduced to the porphyrin by starting with more elaborate building blocks containing esters. Classic carboxylated *meso*-aryl porphyrins, such as *meso*-tetrakis(4-carboxyphenyl)porphyrin, have been prepared by Adler synthesis with ester-substituted benzaldehydes followed by saponification of the esters [40]. Tetracarboxylated palladium porphyrin **62** was prepared thusly, whereby the carboxylate moieties were revealed by standard ester saponification conditions after the palladium insertion step (Scheme 17) [106].

**Scheme 18:**
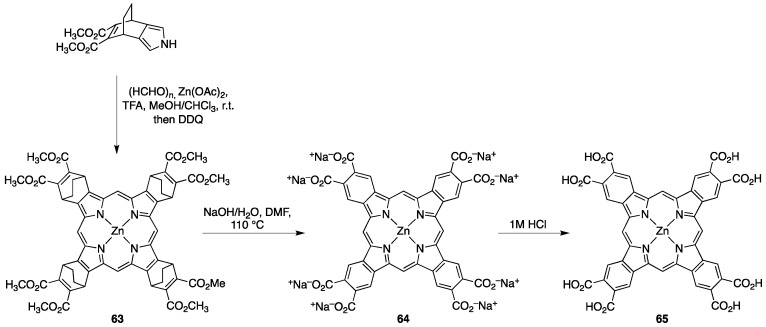
Carboxylated benzoporphyrins, as described by the group of Sugimoto [107].

An introduction of esters to the β-positions of a porphyrin from ester-substituted building blocks is also possible, as illustrated by the synthesis of carboxylated benzoporphyrins starting from an ester-functionalized isoindole (Scheme 18) [107]. A retro-Diels-Alder reaction of **63** with concomitant saponification of the methyl esters afforded octacarboxylate **64**. Protonation provided the octaacid **65** that also precipitated under these conditions. The water-solubility of the sodium salt **64** was determined to be 0.111 M [107].

**Scheme 19:**
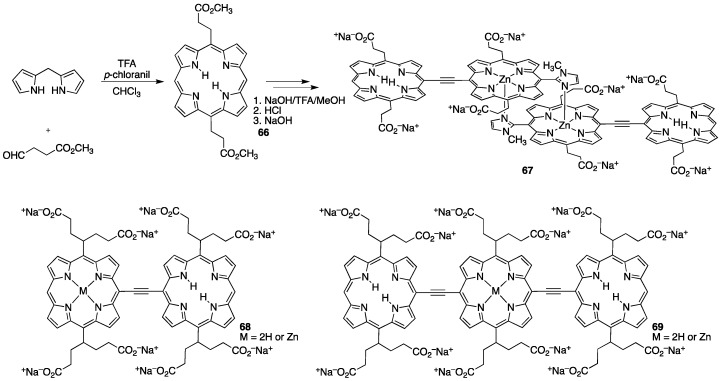
Porphyrin dimers and trimers bearing carboxylate groups in the *meso*-positions, as described by the group of Kobuke [108,109].

Water-soluble acetylene-linked bis(imidazolylporphyrins) were prepared from starting materials bearing protected acid functionalities. Thus, ester-functionalized aldehyde and dipyrromethane were condensed and oxidized to synthesize porphyrin **66** bearing ester-terminated alkyl chains in the *meso*-positions. The ester moieties were carried through the entire synthesis of the porphyrin dimer and the carboxylates were only deprotected in the last step (Scheme 19) [108]. Using this strategy, bulky bis(carboxylethyl)methyl substituents could also be introduced to the *meso*-positions early in the synthesis to furnish dimers of type **68** and trimers of type **69**, which showed less aggregation in water [109].

**Scheme 20:**
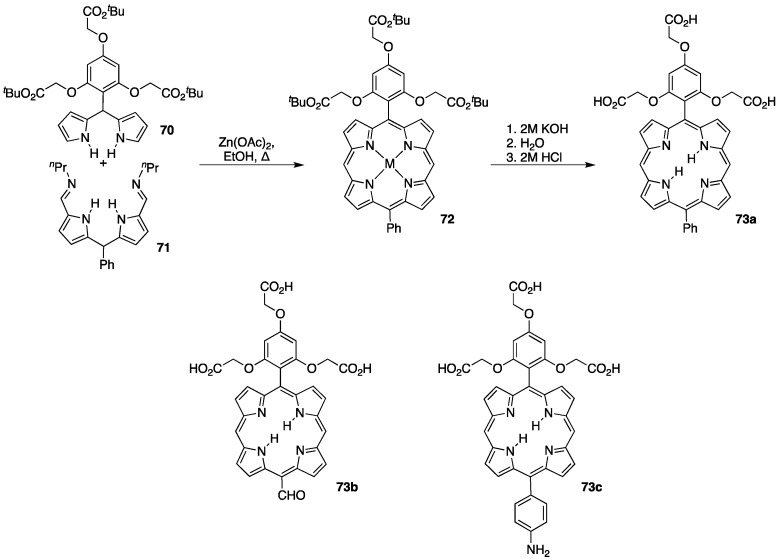
Carboxylated *trans*-AB porphyrins, as described by the group of Lindsey [110].

Lindsey and co-workers prepared carboxylated *trans*-AB porphyrins using a 2 + 2 dipyrromethane condensation strategy, using dipyrromethane **70** carrying a protected 2,4,6-triester-subsituted *meso*-phenyl group (Scheme 20) [110]. The condensation of **70** and counterpart **71** in the presence of zinc afforded the zinc-porphyrin triester **72**, which could be saponified to afford the tricarboxylic acid **73a**. Along an analogous route, *trans*-AB porphyrins **73b** and **73c** bearing bioconjugatable groups at the *meso*-position opposite the solubilizing groups were prepared.

**Scheme 21:**
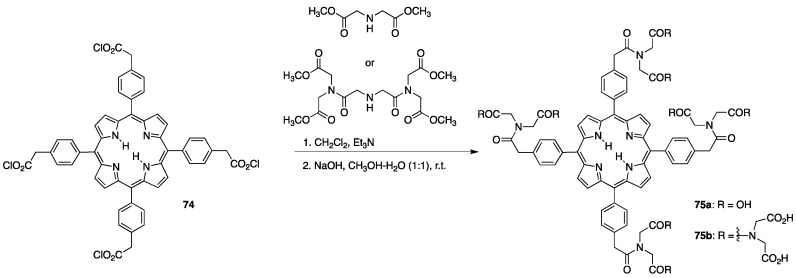
Dendritic carboxylated porphyrins, as described by the group of Mukhtar [111].

To maximize the number of carboxylic acid groups introduced, carboxylic acid ester-terminated dendritic components can be utilized, often bound to the porphyrin through amide linkages. For instance, treatment of tetrarylporphyrin acid chloride **74** with dimethyl iminodiacetate or its second generation dendrimer analogue, followed by saponification of the esters, afforded the polyacid porphyrins **75a** or **75b**, respectively (Scheme 21) [111]. They were purified by precipitation and filtration.

1,4,7,10-Tetraazacyclododecane-1,4,7,10-tetraacetic acid (DOTA) groups were also introduced to porphyrins through amide linkers on one or more *meso*-aryl groups of a porphyrin. These compounds were used as multimodal heterometallic complexes for MRI/PET imaging applications [112,113].

*meso*-Tetrakis(4-hydroxyphenyl)porphyrin (**76**) is a versatile starting material for the synthesis of hydrophilic derivatives since the *p*-phenolic oxygens can react with a variety of solubilizing groups. A_4_-Porphyrin **76** is synthesized through the Adler procedure, generally as its 4-methoxy derivative, that can be purified and handled easily. It is subsequently deprotected using BBr_3_ [14] (see also below, Scheme 23 and Scheme 47).

**Scheme 22:**
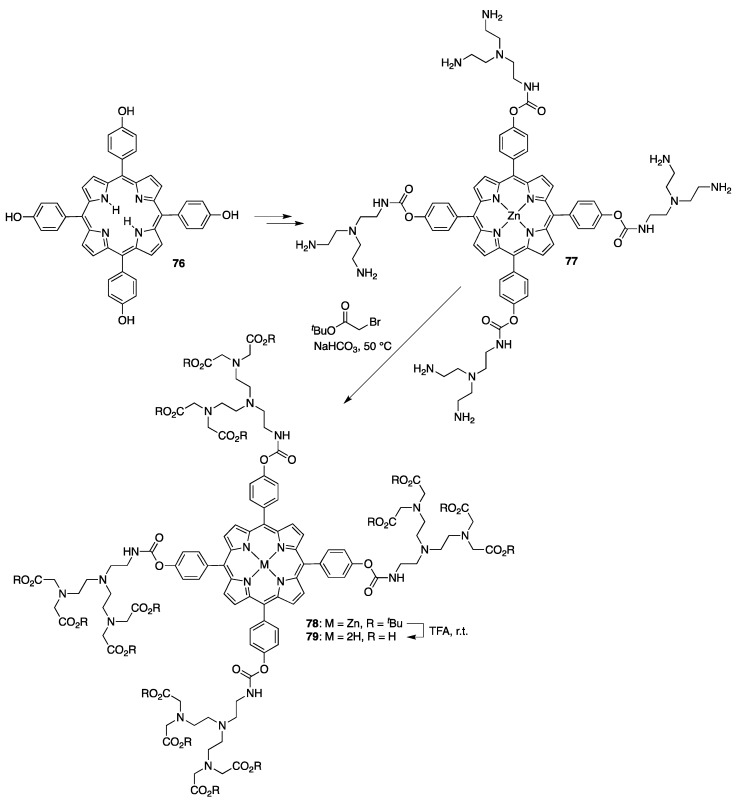
Dendritic polycarboxylated porphyrins, as described by the group of Chen [114].

Using tetraphenol **76**, Chen and co-workers also synthesized porphyrins bearing dendritic carboxylate groups, albeit their synthesis was less convergent than that of Mukhtar and co-workers [114], since the dendrimer generations were built up on the porphyrin core. Thus, **76** was converted to polyamine-derivatized porphyrin **77** in three steps. Reaction of the amines in **77** with bromoacetic acid ethyl ester provided the protected porphyrin **78**, followed by hydrolysis with TFA to give the free acid **79** (Scheme 22) [114] that was isolated by precipitation (addition of a MeOH solution of **79** to Et_2_O).

**Scheme 23:**
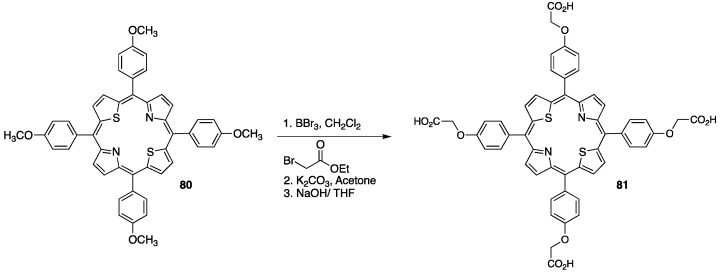
Core-modified carboxylated porphyrin, as described by the group of Detty [115].

Alkylation strategies have also been employed for the introduction of carboxylates. For instance, various carboxylated core-modified porphyrins were prepared by *O*-alkylation of the phenolic oxygens of a *meso*-4-hydroxyphenyl-substituted core-modified dithiaporphyrin. Like their aza-analogues, the requisite core-modified porphyrins were synthesized with a methyl-ether protecting group in the *p*-position of one or more phenyl groups and deprotected using the standard BBr_3_ method. The phenolic oxygens were then alkylated with ethyl bromoacetate. Saponification of the installed esters furnished the dithiaporphyrin **81** bearing carboxylic acid functional groups (Scheme 23) [115].

Diethylenetriamine-*N*,*N*,*N*′′,*N*′′-tetraacetate (DTTA) groups were also introduced to *meso* positions of a porphyrin by alkylation of *meso*-tetrakis(bromomethylphenyl)porphyrin [116]. The DTTA groups were introduced as protected *t*-butyl esters and deprotected using standard conditions. The water-soluble products were purified by sequential cation and anion exchange chromatography.

**Scheme 24:**
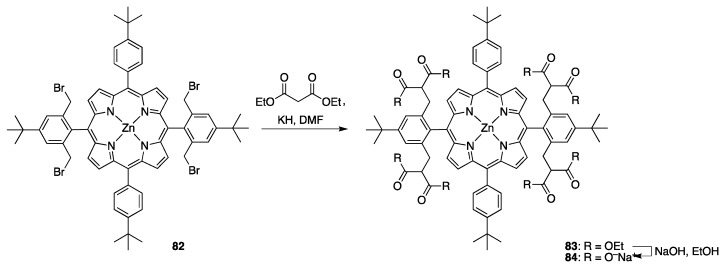
Octacarboxylated porphyrin **84**, as described by the group of Prato [97].

Prato and co-workers also used an alkylation strategy to synthesize octacarboxylated porphyrin **84**, starting from a tetra(bromomethyl)-substituted porphyrin. Alkylation of porphyrin **82** with diethyl malonate afforded the tetramalonic ester-derivatized porphyrin **83**, which was saponified to free the eight carboxylate moieties (Scheme 24) [97]. Octacarboxylate **84** precipitated under the saponification conditions and was isolated by filtration to afford the pure product.

**Figure 6:**
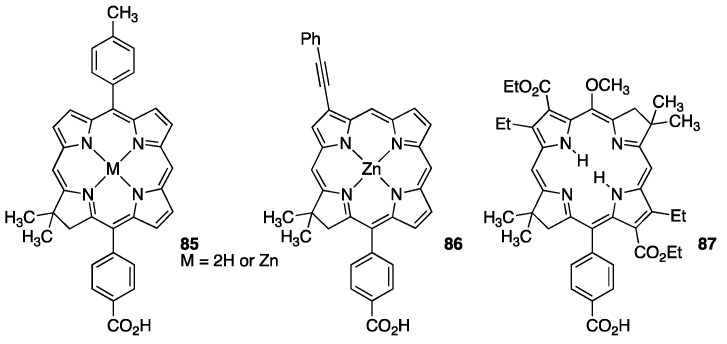
Molecular structures of *meso*-carboxyphenyl-substituted chlorins **85** and **86**, and bacteriochlorin **87**.

Carboxyphenyl substituents can also be directly introduced to the *meso*-positions of synthetic chlorins and bacteriochlorins by Suzuki coupling of suitable *meso*-bromo-substituted porphyrins with boronic esters derived from benzoic acid. Using the same Suzuki coupling approach that was used to introduce pyridyl substituents to the *meso*-position (cf. Scheme 12), chlorins **85**, **86** and the bacteriochlorin **87** (Figure 6) were prepared [91]. The carboxylate functionality was introduced in the free acid form, eliminating the need for the utilization of protecting groups.

**Scheme 25:**
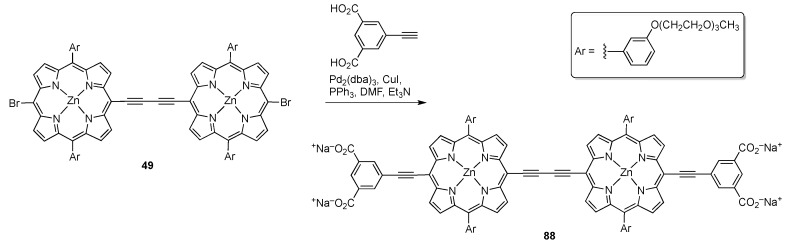
Carboxylated bis-acetylene linked porphyrin dimers, as described by the group of Anderson [70]. dba = dibenzylacetone.

As well starting from *meso*-brominated precursors, the group of Anderson used a Sonogashira coupling strategy to functionalize the *meso*-positions of a PEGylated porphyrin dimer with carboxylate groups. *meso*-Dibromoporphyrin dimer **49** was coupled with 5-ethynyl-1,3-benzenedicarboxylic acid to form the tetracarboxylated water-soluble porphyrin dimer **88** (Scheme 25) [70]. Again, it is noteworthy that the acid functionality was introduced in unprotected form.

**Scheme 26:**
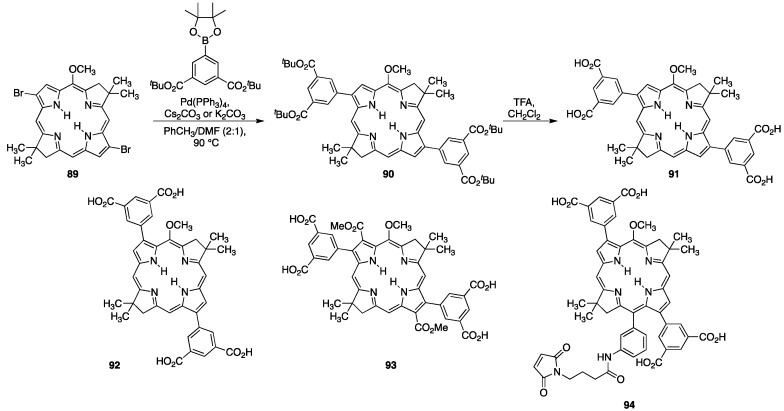
Tetracarboxybacteriochlorins, as described by the group of Lindsey [117].

Functionalization of the β-position of bacteriochlorins with carboxyphenyl groups was also achieved by Suzuki coupling, albeit this method required the use of protected boronic ester carboxylates [117]. Thus, β-dibromoporphyrin **89** was functionalized using the boronic ester of the *t*-butyl ester-protected 3,5-carboxyphenyl substituents (Scheme 26). The protecting groups were removed at the last step of the synthesis by acid treatment. Furthermore, the option for bioconjugation was demonstrated in these systems by Suzuki coupling of a *meso*-bromobacteriochlorin, generated by regioselective bromination of the parent bacteriochlorin, to produce hydrophilic tetracarboxyl-bacteriochlorin **94** [117].

### 3.2. Sulfonated Porphyrins

Sulfonation of *meso*-tetraphenylporphyrin proceeds regioselectively at the *para*-positions of the *meso*-phenyl rings [118]. This reaction led to the earliest examples of water-soluble *meso*-tetraaryl-porphyrins [40]. A number of sulfonated A_4_ porphyrins were prepared by direct sulfonation methods [40], and are commercially available [58]. However, this solubilization strategy has drawbacks, not the least of which are the harsh reaction conditions (conc. H_2_SO_4_, ∆) required for the sulfonation step, and the difficulty in purifying the target compound. On the other hand, sulfonation readily leads to the solubilization of porphyrins in aqueous media. Interestingly, the porphyrin is protonated during the sulfonation step and is thus protected by the dicationic charge from getting sulfonated at the β-positions along an electrophilic aromatic substitution pathway, in contrast with the regioselective β-sulfonation of the less basic corroles using chlorosulfonic acid [119,120,121].

One particular challenge faced by using sulfonation procedures is the incorporation of other functional groups since they may be incompatible with the harsh sulfonation conditions. The sulfonation step is often employed directly after porphyrin formation, then subsequent modification of the porphyrin is performed. The drawback of this strategy is the difficulty in handling and purification of the sulfonated porphyrins.

**Scheme 27:**
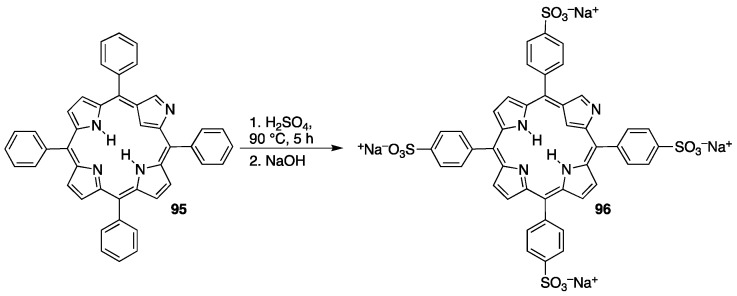
Sulfonated *N*-confused porphyrin, as described by the group of Pillai [122].

Despite these disadvantages, the traditional direct sulfonation method was employed by Pillai and co-workers to regioselectively introduce sulfonate groups to the *meso*-*p*-phenyl positions of *N*-confused porphyrins (Scheme 27) [122]. Sulfonation of *N*-confused porphyrin **95** by treatment with H_2_SO_4_ at elevated temperature over several hours afforded the tetrasulfonated porphyrin **96** that was tested as a photosensitizer for PDT [122]. The product was purified by precipitation and continuous Soxhlet extraction with MeOH. No further functionalizations of this porphyrin derivative were reported.

**Scheme 28:**
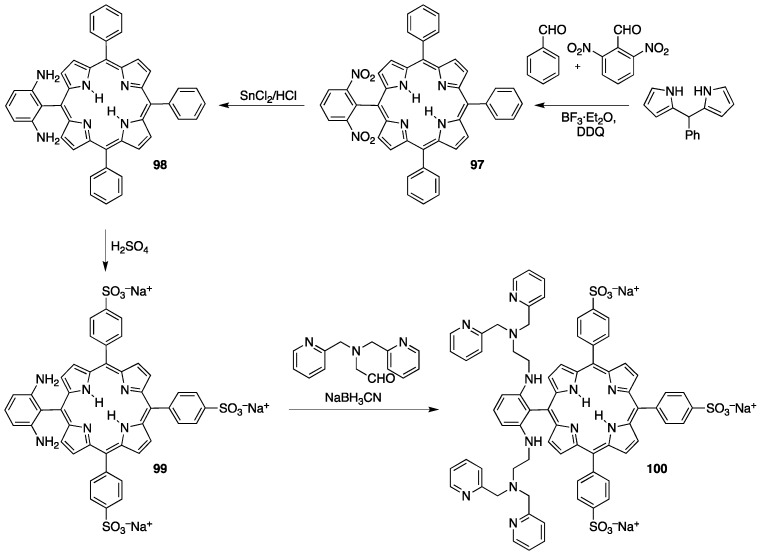
Sulfonated A_3_B porphyrin bearing DPA substituents for fluorescence sensing of Zn^2+^, as described by the group of Lippard [124].

Likewise, asymmetric *meso*-aryl porphyrins were also subjected to sulfonation reactions. For instance, A_3_B porphyrin *meso*-trisphenyl-4-pyridylporphyrin, made by mixed aldehyde condensation, could be trisulfonated using oleum at 80 °C [123]. The pure product was obtained by precipitation and centrifugation after neutralization of the acid. The pyridyl moiety could then be functionalized with a platinum complex for study as a tumor targeting photosensitizer. The group of Lippard prepared the A_3_B porphyrin *meso*-trisphenyl-(2,6-nitrophenyl)porphyrin **97** (Scheme 28). Reduction of the nitro group afforded the 2,6-diaminophenyl porphyrin **98** that was subjected to sulfonation. The water-soluble product **99** was purified and isolated by reverse phase chromatography (RP-18). The purified free amine could then be functionalized with DPA for its use as a Zn^2+^ chemosensor [124]. Furthermore, the manganese complex of **100** was prepared and used as a dual function contrast agent for the fluorescence imaging of Zn^2^ and MRI in biological contexts [124].

**Scheme 29:**
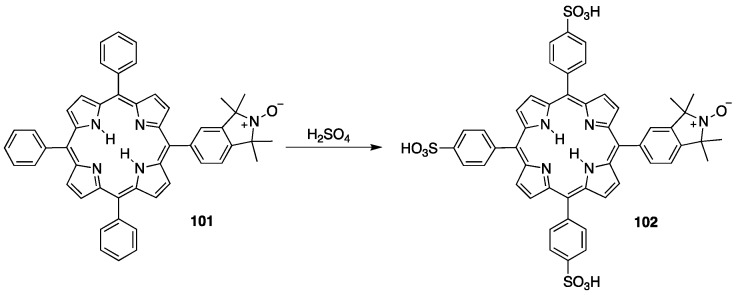
Sulfonated isoindoline nitroxide-functionalized A_3_B porphyrin, as described by the group of Yang [125].

As the previous examples have shown, some functionalities tolerate the sulfonation conditions. Surprisingly, this is also the case for isoindoline nitroxide-functionalized A_3_B porphyrin **101**. All phenyl substituents could be sulfonated by treatment with H_2_SO_4_ with retention of the isoindoline nitroxide (Scheme 29), albeit the yield for the water-soluble final product **102** was low (~30%) [125].

**Scheme 30:**
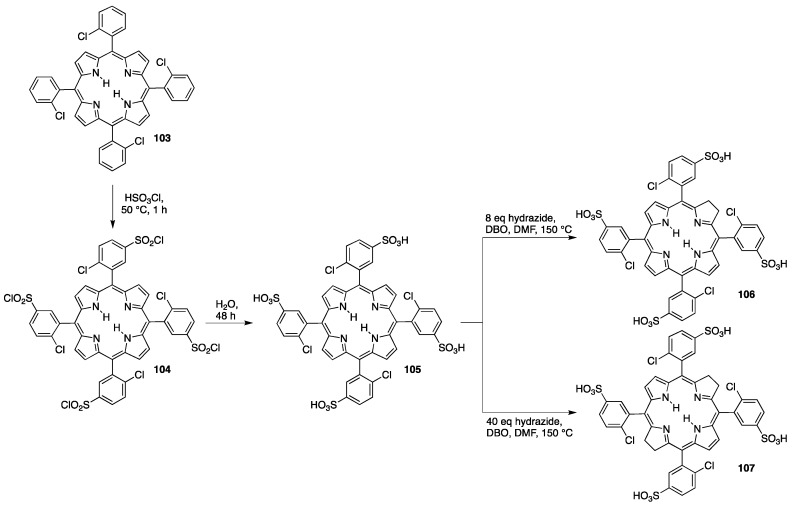
Chlorosulfonated porphyrins and their hydrolysis and reduction products, as described by the group of Wyatt [126]. DBO = 1,4-diazobicyclo[2.2.2]octane.

A chlorosulfonation method has been used for the introduction of sulfonic acid groups to a number of halogenated *meso*-aryl porphyrins by the group of Wyatt and co-workers (Scheme 30) [126]. Treatment of chlorinated porphyrin **103**, for example, with chlorosulfonic acid afforded chlorosulfonation product **104** that was hydrolyzed by suspension in H_2_O to provide the corresponding sulfonic acid product **105**. Alternatively, the chlorosulfonate could also be converted to sulfonamide derivatives by reaction with amines [126]. The corresponding chlorin and bacteriochlorin **106** and **107**, respectively, were made by diimide reduction of the sulfonated porphyrin **105**, and were tested as photosensitizers for the PDT of tumors (Scheme 30) [127].

**Scheme 31:**
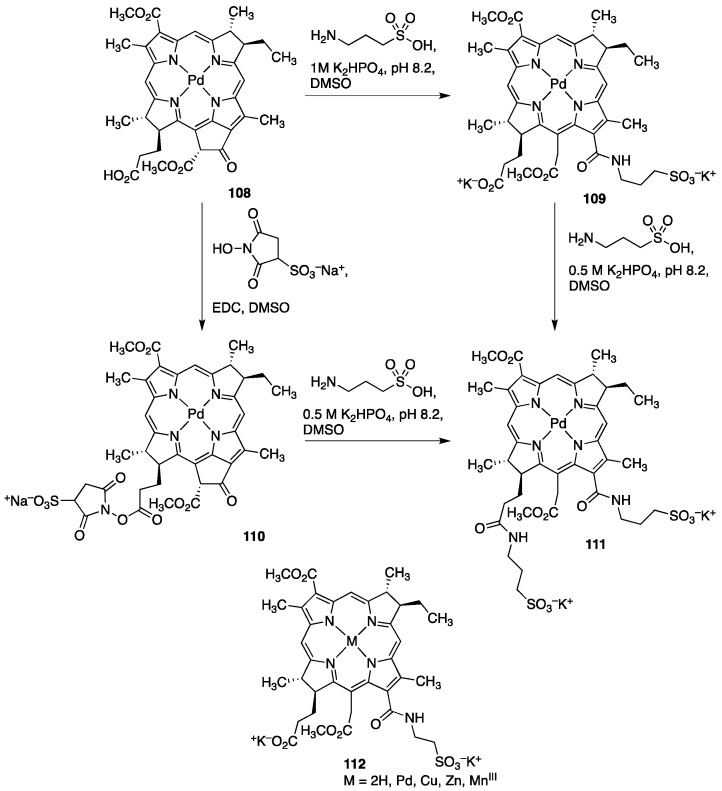
Sulfonated Pd-bacteriopheophorbide, as described by the group of Scherz [129]. EDC = 1-ethyl-3-(3-dimethylaminopropyl)carbodiimide.

The attachment of sulfonated groups to the β-positions of a tetrapyrrolic macrocycle is an alternative method toward the generation of hydrophilic sulfonated derivatives. This approach was applied to the generation of alkylsulfonated bacteriochlorins [128]. For example, sulfonated bacterio-chlorophyll derivatives were prepared by reaction of Pd-bacteriopheophorbide (**108**) with homo-taurine (Scheme 31) [128]. Treatment of either **109** or the sulfo-NHS ester **110** with a second equivalent of homotaurine also yielded the bis-sulfonated product **111** (with the trisulfonated compound arising from imine formation with the β-acetyl group as a minor product). Bacteriochlorin **112M** could also prepared using a similar procedure using taurine as the nucleophile. All of the sulfonated derivatives were found to possess high solubility in water (up to 40 mg/mL) [129]. The products were either purified by HPLC (MeOH/phosphate-buffer solvent systems) or by silica gel chromatography (4:1 CHCl_3_:MeOH).

**Figure 7:**
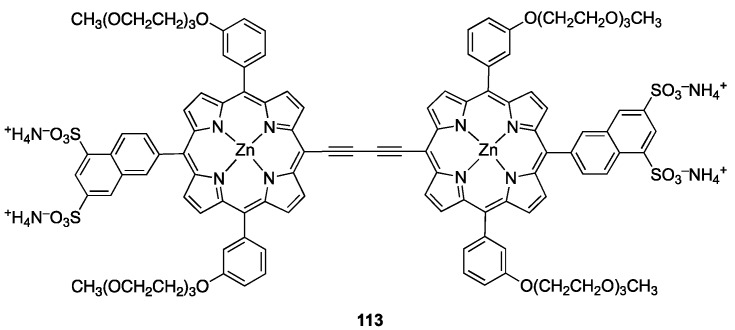
Molecular structure of sulfonated biporphyrin **113**.

Sulfonate functionalities may also be introduced at a late stage in the porphyrin synthesis by transition metal-mediated cross-coupling strategies. For instance, Anderson and co-workers introduced sulfonate groups to the *meso*-positions of a *meso*-brominated PEGylated conjugated porphyrin dimer using a Suzuki coupling with a sulfonated arylboronic acid, a strategy similar to the introduction of pyridine functionalities described above (cf. Scheme 13) [70]. The tetrasulfonated porphyrin dimer **113** (Figure 7) was purified using semi-preparative reverse-phase HPLC.

### 3.3. Porphyrins with Phosphate/Phosphonate Functional Groups

The introduction of alkylphosphonate functional groups to the periphery of a porphyrin is another strategy to impart hydrophilicity to porphyrins and hydroporphyrins. Phosphate groups have also been employed, but because of the increased hydrolytic lability of the phosphate group, only rarely.

**Scheme 32:**
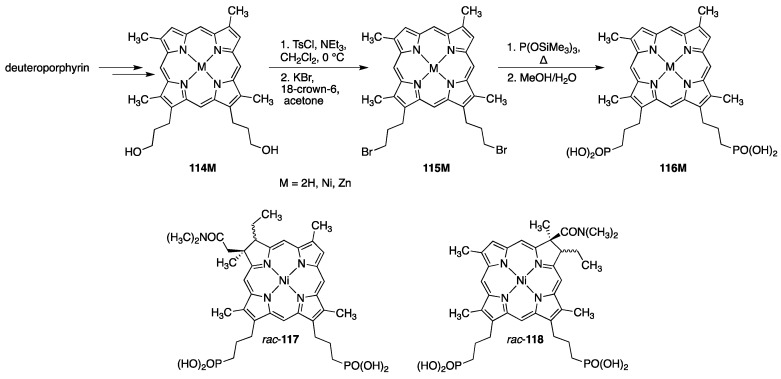
Phosphonated β-alkyl porphyrin derivatives, as described the group of Montforts [130].

Alkyl phosphonates have been introduced to the porphyrin by modification of a porphyrin or by the synthesis of the porphyrin macrocycle with protected phosphonate/phosphate or alcohol building blocks. Deprotection and subsequent modification of the porphyrin furnished the water-soluble phosphonated derivatives. One of the first examples of porphyrins bearing phosphonate groups was introduced by the group of Montforts [130]. They prepared water-soluble metalloporphyrins by phosphonation of the primary alkyl alcohols in deuteroporphyrin derivative **114M** (Scheme 32) [130]. Their strategy involved the conversion of the alcohols in **114M** to brominated derivative **115M**, followed by reaction with P(OSiMe_3_)_3_, and subsequent hydrolysis of the intermediate with MeOH/H_2_O to afford phosphonate **116M**. The free phosphonate functional groups were used to attach these derivatives to electrode surfaces [130]. Using a similar strategy, diphosphonated chlorin derivatives ***rac*-117** and ***rac*-118** were also prepared (Scheme 32).

**Scheme 33:**
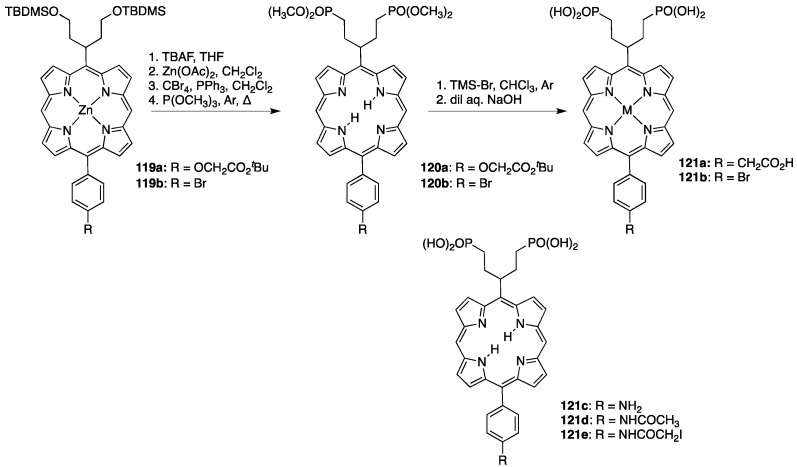
*trans*-AB Porphyrins bearing swallowtail phosphonate motifs, as described by the group of Lindsey [131,132].

While the requisite alkyl alcohols for phosphonation in **114M** were generated by functional group manipulation of the propionic acid side chains present in the natural product precursors, the requisite alcohols can also be introduced (in free or protected form) at the porphyrin synthesis stage. For instance, Lindsey and co-workers prepared *trans*-AB porphyrin **119** by using a dipyrromethane precursor bearing two *t*-butyldimethylsilyl-(TBDMS) protected hydroxyl groups (Scheme 33) [131]. After removal of the protecting groups, the hydroxyl groups could be derivatized in a similar manner as described by Montforts [130] to provide the protected phosphonate ester **120**. An ester hydrolysis revealed the free phosphonate groups, conveying water-solubility. The corresponding zinc complexes were also prepared.

**Scheme 34:**
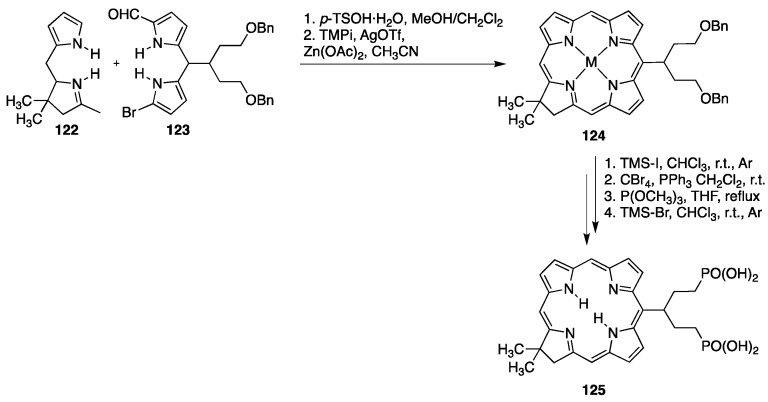
Synthetic chlorins bearing phosphonate swallowtail motifs, as described by the group of Lindsey [133]. TMPi = 2,2,6,6-tetramethylpiperidine.

Along analogous pathways, *trans*-AB porphyrins bearing amino (**121c**), acetamido (**121d**), and iodoacetamido (**121e**) bioconjugatable tethers at the *meso*-phenyl position opposite the solubilizing group also became accessible (Scheme 33) [132]. This approach is suitable for the synthesis of phosphonated chlorins, synthesized by using dipyrromethane building block **123**, bearing two protected alcohols (Scheme 34) [133]. After condensation of **122** and **123**, the benzyl ether protecting groups in **124** were removed, and the alcohols were brominated and phosphonated. The phosphonate ester was then treated with TMS-Br to furnish the free phosphonates.

**Scheme 35:**
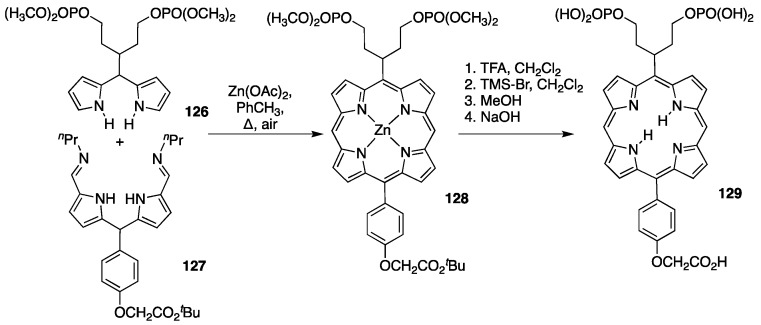
Porphyrin bearing phosphate swallowtail motif, as described by the group of Lindsey [131].

Lindsey and co-workers also prepared *trans*-AB porphyrins incorporating phosphate groups, though in this instance they started with a dipyrromethane precursor already bearing phosphate esters (Scheme 35) [131]. Hydrolysis of the protected phosphate groups afforded the free phosphate groups.

**Figure 8:**
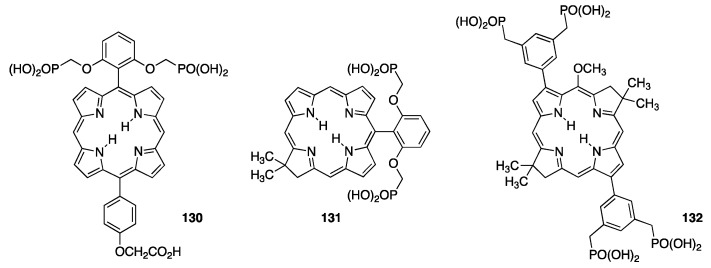
Molecular structures of phosphonated porphyrin **130**, chlorin **131**, and bacteriochlorin **132**.

Phosphonate groups can also be introduced to the porphyrin by starting with dipyrromethane precursors bearing protected phosphonate functional groups. Using this strategy, *trans*-AB porphyrin **130** (Figure 8) was synthesized by condensation using a dipyrromethane precursor carrying an 2,6-diphosphonated aryl ring that projects the solubilizing groups above and below the plane of the macrocycle target (cf. Scheme 35) [134]. Hydrolysis of the phosphonate esters and saponification affords the *trans*-AB porphyrin bearing both the phosphonate solubilization groups and a carboxylic acid bioconjugatable tether. Using a similar strategy as for chlorin **125**, this 2,6-aryl-phosphonate motif was also incorporated into chlorin 131 [133]. Again, the Suzuki coupling strategy of β-dibromobacteriochlorins proved versatile enough to be applicable to the introduction of β-aryl-phosphonate groups (cf. Scheme 6) to afford, after hydrolysis of the phosphonate esters, water-soluble bacteriochlorin **132**.

## 4. Water-Soluble Porphyrins with Neutral Groups

### 4.1. PEGylated Porphyrins

PEGylation has emerged as a strategy for the preparation of neutral water-soluble porphyrins and hydroporphyrins. It has many benefits over traditional means using ionic groups. For instance, PEGylation strategies are more flexible with respect to where in the synthetic sequence they are introduced. This is because the PEG functional group is not very reactive once installed. Unwanted reactivity to form oligomeric species is also suppressed by using heterobifunctional PEG chains, in which one end of the PEG chain often has a methyl cap. PEGylated porphyrins are also much more amenable to traditional aqueous work-up conditions, as they may partition favorably in organic solvents such as CH_2_Cl_2_ and EtOAc [135,136]. Often times PEGylated porphyrins can be purified by normal silica-gel chromatography using polar solvents [14,137,138,139].

#### 4.1.1. Porphyrinoid Macrocycle Total Synthesis Using PEGylated Building Blocks

**Scheme 36:**
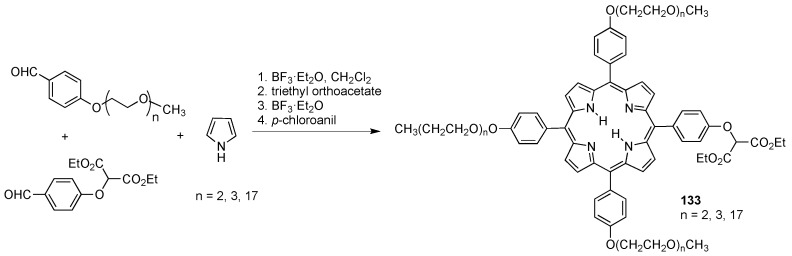
A_3_B porphyrin carrying three short PEG chains, as described by the group of Brunner [140].

Despite these advantages, the multistep manipulation of porphyrins with PEG-groups present throughout the synthesis is relatively rare. For instance, Brunner and co-workers presented such an example. They synthesized the A_3_B porphyrin **133** by mixed condensation of two arylaldehydes and pyrrole, with one of the aryl aldehydes carrying a PEG chain (*n* = 2, 3 or 17) (Scheme 36) [140]. The resulting A_3_B PEGylated porphyrins **133a**–**133c** were purified using ordinary silica gel chromatography (CH_2_Cl_2_/MeOH mixtures). The PEGylated porphyrins were also amenable to further modification at the malonic ester functional group to generate platinum-porphyrin conjugates.

**Scheme 37:**
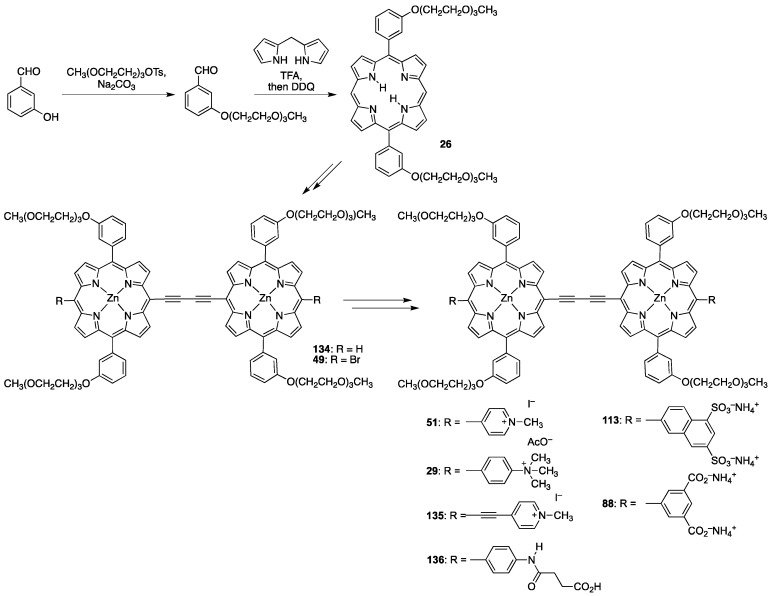
Hydrophilic bis-acetylene linked porphyrin dimers carrying PEG chains and various other solubilizing motifs, as described by the group of Anderson [70].

Another example for the early introduction of PEGylated building blocks was reported by the group of Anderson [70]. Using a PEGylated benzaldehyde, porphyrin **26** bearing two methyl-capped tetraethylene glycol (TEG) groups on opposite *meso*-phenylgroups was prepared. Leaving the PEG chains intact, it was converted to the bis-acetylene linked porphyrin dimer **134**. Porphyrin dimer **134** was found not to be sufficiently soluble in aqueous media for biological application. Therefore, these materials were further derivatized with additional solubilizing groups, all while the PEG functional groups remained intact (Scheme 37) [70].

Starting from the *meso*-dibromo dimer **49**, cationic (pyridinium; cf. Scheme 13), anionic (sulfonate) and carboxlyate (cf. Scheme 25) groups were introduced using either Suzuki or Sonogashira coupling strategies. The Senge arylation method was also used to introduce ammonium and pyridyl solubilizing groups (cf. Scheme 7). The dimers were separated by either recrystallization (DMF, Et_2_O) or by semi-preparative reverse phase HPLC. Due to insufficient solubility of the compounds in regular reverse-phase HPLC solvents (such as CH_3_CN, MeOH, water), DMF, DMSO, or THF were used. Also due to solubility issues, the authors could not measure logP values for these compounds in octanol:water. However, they did compare the relative polarity of some derivatives by means of their retention times under reverse-phase HPLC conditions (Table 1).

**Scheme 38:**
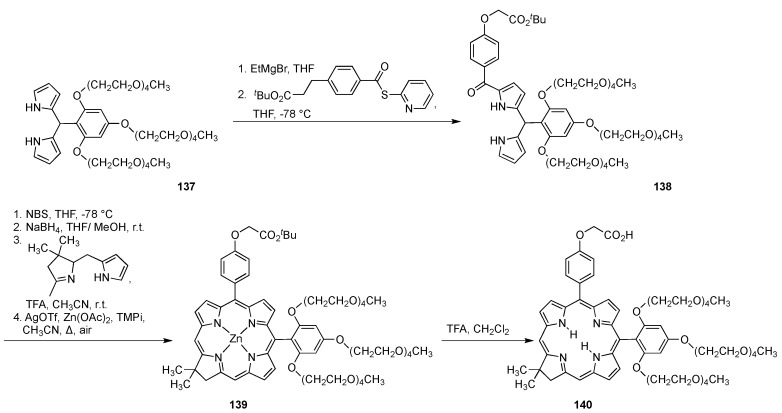
PEGylated synthetic chlorins using PEGylated building blocks, as described by the group of Lindsey [136]. TMPi = 2,2,6,6-tetramethylpiperidine.

PEG-substituted dipyrromethanes also found use in the early introduction of solubilizing groups. The group of Lindsey was able to synthesize chlorins from a PEGylated dipyrromethane precursor (Scheme 38) [136]. This strategy also incorporated a bioconjugatable tether at another *meso*-position, the precursor of which was also introduced at the dipyrromethane stage.

#### 4.1.2. PEGylation of *meso*-aryl Porphyrins

A number of *meso*-tetraarylporphyrins bearing PEG groups have been synthesized by PEGylation of porphyrins incorporating suitable linker functionalities on the aryl groups. These porphyrins are usually prepared via Adler or Lindsey methods and the PEG groups can be linked by various methods including etherification, amidation or esterification reactions. Particularly popular linking groups for the PEGylation of porphyrins are *meso*-phenol substituents. PEGylation of various *meso*-tetrahydroxyphenyl porphyrin derivatives with a suitably functionalized PEG precursor yields water-soluble porphyrins, with activation of the PEG group often required.

**Scheme 39:**
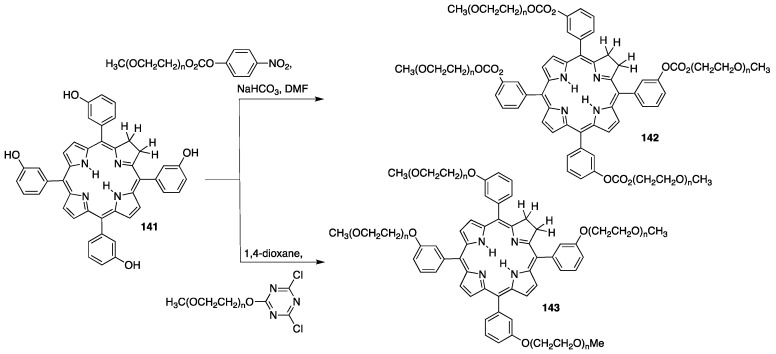
PEGylated derivatives of *meso*-tetrakis(3-hydroxyphenyl)chlorin, as described by the group of Sinn [141].

For example, *meso*-tetrakis(3-hydroxyphenyl)chlorin (**141**) was rendered water-soluble by introducing PEG groups through two different etherification strategies. Tetraphenolporphyrin **141** was treated with *p*-nitrophenylcarbonate- or cyanuric chloride-activated methoxypolyethyleneglycol to afford the water-soluble PEGylated porphyrins **142** or **143**, respectively (Scheme 39) [141].

**Scheme 40:**
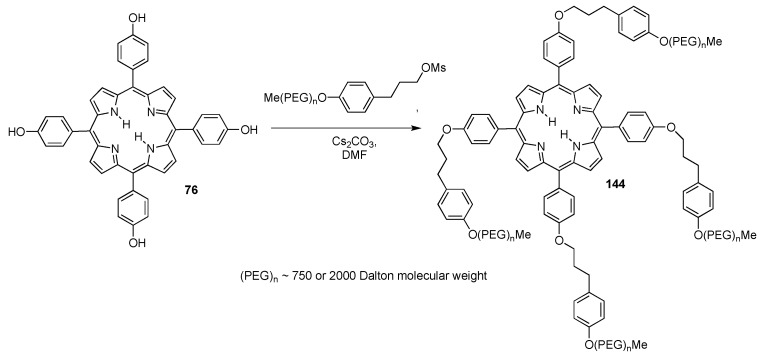
PEGylated derivatives of *meso*-tetra(4-hydroxyphenyl)porphyrin, as described by the group of Pozzi [137].

*meso*-Tetrakis(4-hydroxyphenyl)porphyrin (**76**) can also be alkylated using various activated PEGs. For instance, this procedure has been employed to alkylate **76** by treatment with PEG-mesylates to form **144**. (Scheme 40) [137].

**Scheme 41:**
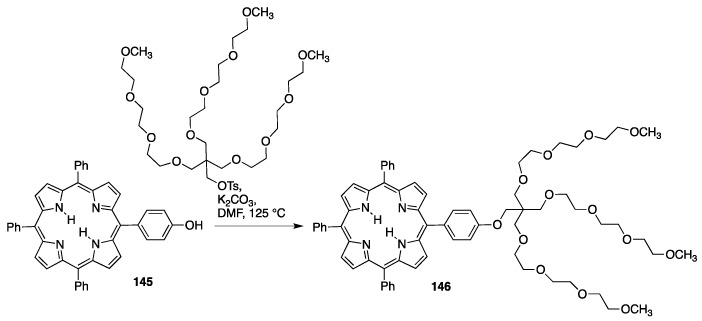
Amphiphilic porphyrin with a PEG-dendrimer, as described by the group of Dumoulin [142].

Under similar conditions, single PEG chains or PEG-dendrimers (Scheme 41) [142] can also be introduced into asymmetric *meso*-hydroxyphenylporphyrins [143,144]. Vitalini and co-workers prepared PEGylated derivatives of **76** by alkylation of the phenolic oxygens with a chlorinated PEG [145]. The PEG-functionalized porphyrins could be purified by adding a solution of the compound in CH_2_Cl_2_ to a cold solution of Et_2_O to induce precipitation (for larger PEGs) [137,143]; porphyrins bearing smaller PEG-chains could also be purified by column chromatography (CH_2_Cl_2_-acetone-MeOH or CH_2_Cl_2_/EtOH mixtures) [137,142].

**Scheme 42:**
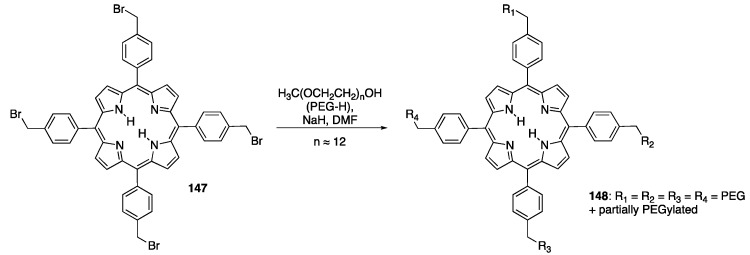
PEGylated *meso*-aryl porphyrins by benzylation of a PEG with tetra(*p*-bromomethylphenyl)porphyrin, as described by the group of Lee [146].

PEGylation of *meso*-arylporphyrins was also achieved by inversion of the Williamson ether synthesis reaction partners, i.e., involving a halogenated porphyrin precursor and PEGs bearing a free alcohol. An example is the PEGylation of tetra(*p*-bromomethylphenyl)porphyrin (**147**) with a methyl-capped PEG alcohol (M_n_ = 550) that afforded the tetra-PEGylated porphyrin **148** as the main product, along with partially PEGylated porphyrins (Scheme 42). The porphyrin starting material was prepared by Lindsey porphyrin synthesis from 4-(bromomethyl)benzaldehyde and pyrrole [146]. The PEGylated products were amenable to aqueous work-up (extraction with CH_2_Cl_2_) and were purified by HPLC.

**Scheme 43:**
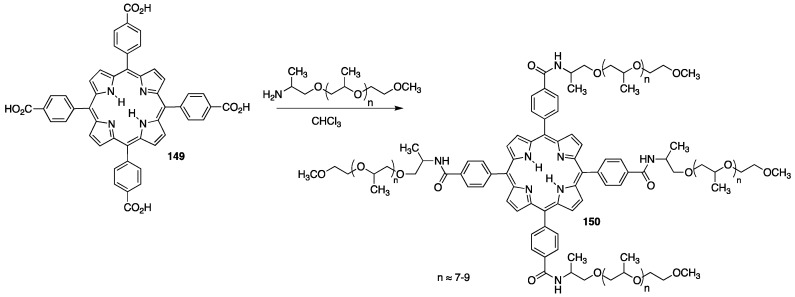
PEGylated porphyrins derived from *meso*-tetrakis(4-carboxyphenyl)porphyrin using a PEG-amine, as described by the group of Scolaro [147].

*meso*-Aryl porphyrins bearing peripheral carboxylic acid functional groups can be PEGylated by amide formation using an amine-terminated PEG. For instance, Scolaro and co-workers prepared PEGylated derivatives of *meso*-tetrakis(4-carboxyphenyl)porphyrin (**149**) by treatment with Jeffamine M-600 (*n* = 7–9) (Scheme 43) [147].

**Figure 9:**
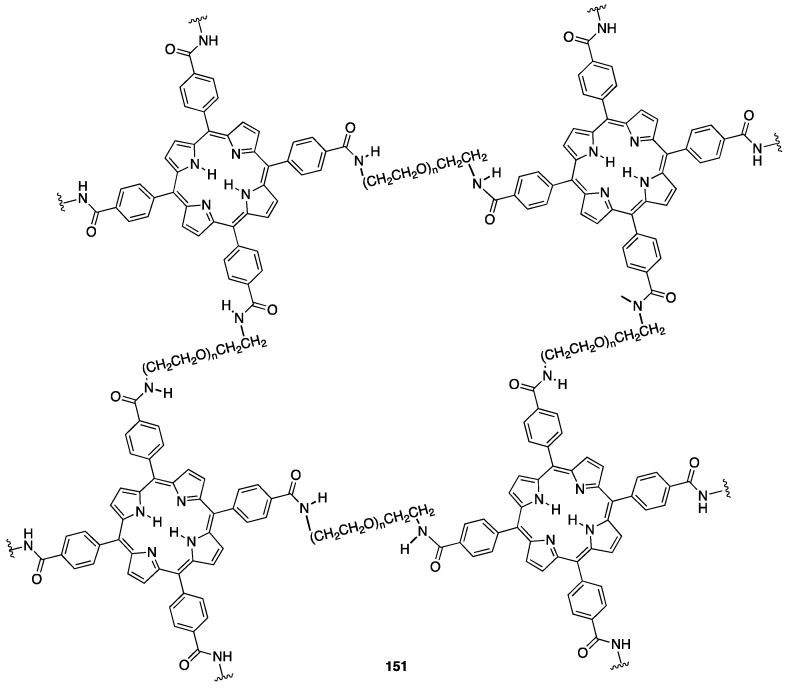
Partial molecular structures of PEG-linked porphyrin mesh **151**.

Also starting from tetraacid **149**, Lovell and co-workers reacted it with a variety of homobi-functional PEG-diamines (average Mw ranging from 150 Da to 10 kDa) to synthesize a PEG-linked porphyrin mesh **151** (Figure 9) [148]. This reaction illustrates the complex cross-linked structure formed when using PEGs capable of reacting at both ends. The porphyrin-PEG-mesh was purified by dialysis, followed by addition of citric acid to precipitate unreacted porphyrin.

PEGylated porphyrins and hydroporphyrins with amide linkages can also be prepared starting with aminophenyl porphyrins. For instance, Peng et al. functionalized a chlorin with a poly-(ε-caprolactone)-polyethyleneglycol) diblock copolymer [149]. Introduction of the PEG-polymer was achieved by treatment of *meso*-tetrakis(4-aminophenyl)chlorin with the acid chloride of the diblock copolymer. The polymer-functionalized porphyrin was purified by precipitation with Et_2_O, followed by a dialysis step in acetone. Vicente and co-workers prepared water-soluble porphyrin-peptide conjugates with a PEG linker on one *meso*-aryl group starting from an AB_3_ porphyrin **152** bearing one *p*-aminophenyl group.

**Scheme 44:**
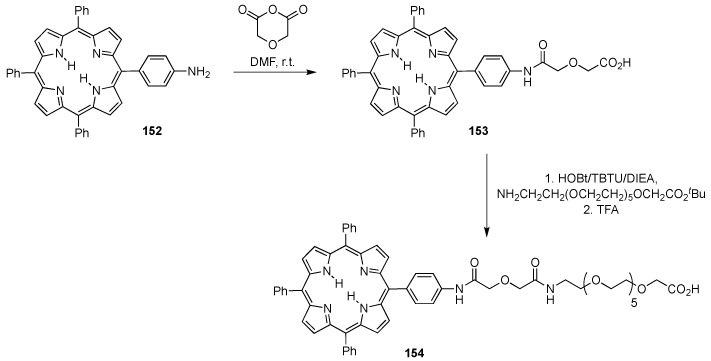
PEGylated AB_3_ porphyrin with carboxylic acid bioconjugation handle, as described by the group of Vicente [150]. HOBt = 1-hydroxybenzotriazole, TBTU = *O*-(benzotriazol1-yl)*N*,*N*,*N*′,*N*′-tetramethyluronium tetrafluoroborate, DIEA = *N*,*N*-diisopropylethylamine.

The amine functionality in **152** was reacted with diglycolic anhydride, followed by amide-coupling using a PEG-amine and Boc-deprotection (Scheme 44). A peptidyl resin can be coupled to the hydroxybenzotriazole (HOBt) ester of the porphyrin **154** [150]. More hydrophilic PEGylated A_3_B porphyrins bearing three carboxyphenyl groups were also prepared in a similar manner [150]. Oligo-ethylene glycol amide-linkages have also been utilized as hydrophilic spacers in instances that require designed structures on the periphery of a hydrophilic porphyrin. Using this strategy, Alessio and co-workers have prepared ruthenium-porphyrin conjugates with oligoethylene glycol spacers [151]. This methodology has also been used to develop Rhenium (I)-porphyrin conjugates in a similar manner [152].

**Scheme 45:**
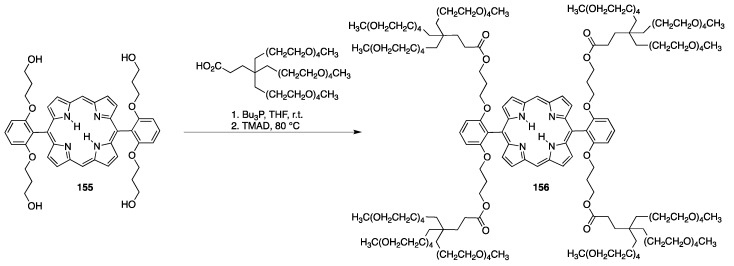
Porphyrin-PEG dendrimer conjugates, as described by the group of Diederich [153]. TMAD = *N*,*N*,*N*′,*N*′-tetramethylazodicarboxamide.

Lastly, PEGylation of *meso*-hydroxyphenylporphyrins can be achieved utilizing ester linkages. Thus, Diederich and co-workers prepared dendritic porphyrin **156** by esterification of porphyrin tetraol **155** with a carboxylic acid-functionalized dendron under Mitsunobu conditions (Scheme 45) [153].

**Scheme 46:**
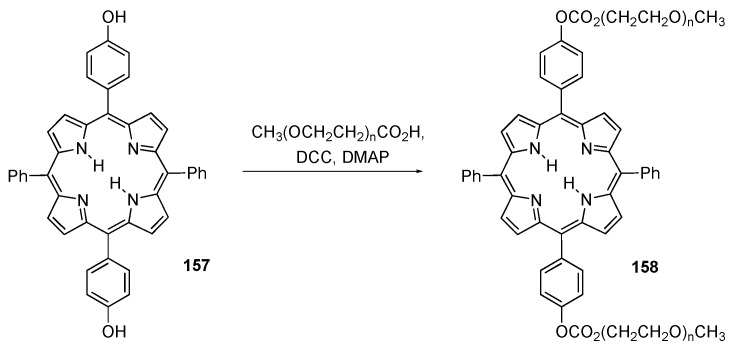
PEGylated A_2_B_2_ porphyrin, as described by the group of Nawaz [154]. DCC = dicyclo-hexylcarbodiimide, DMAP = 4-dimethylaminopyridine.

The extension of the reactive alcohol site from the phenolic position using an alkyl chain was employed to accommodate the bulky PEG-dendrimer substituents. The resulting porphyrin-dendrimers were purified by preparative gel permeation chromatography and were soluble in phosphate buffer at pH 7. Alternatively, the PEGylation of a *meso*-hydroxphenyl A_2_B_2_ porphyrin by DCC/DMAP-mediated esterification with a PEG-carboxylic acid (1000 Da) was reported (Scheme 46) [154].

**Scheme 47:**
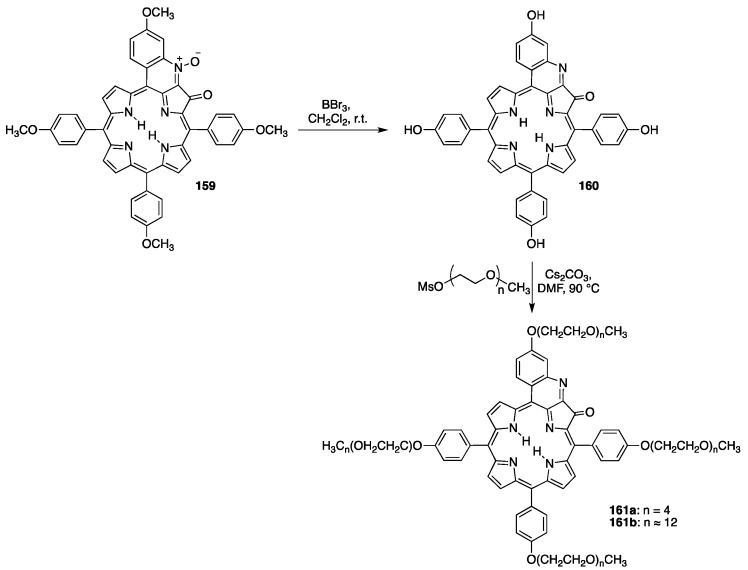
PEGylated quinoline-annulated porphyrins, as described by the group of Brückner [14].

π-Extended porphyrin derivatives may also be subjected to the same PEGylation strategies as seen for regular porphyrins. For instance, PEGylation of the phenolic oxygens in quinoline-annulated porphyrin **160** was reported by Luciano et al. (Scheme 47) [14] using a similar *O*-alkylation method as described for regular porphyrins (Scheme 40).

Precursor porphyrin **159** was prepared from *meso*-tetrakis(4-methoxyphenyl)porphyrin. A BBr_3_-mediated deprotection was employed to deprotect the phenolic oxygens as the penultimate step of the synthesis. The phenol could then be PEGylated using either a short (*n* = 4) or long chain (avg. MW 550, *n* ≈ 12) PEG-mesylate [14]. The short PEG chain derivative **161a** was found to be only slightly soluble in water. However, the derivative bearing long PEGs (**161b**) was soluble in PBS to form ~30 mM solutions and were used as photoacoustic imaging contrast agents for in vivo imaging of tumors in a mouse model [14].

**Scheme 48:**
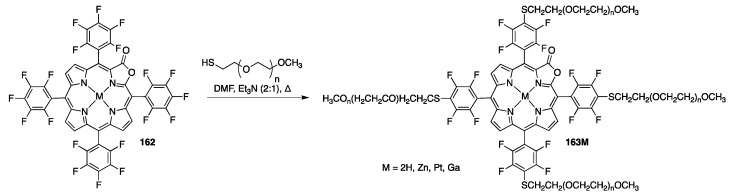
PEGylated porpholactone derived from *meso*-tetrakis(pentafluorophenyl)porphyrin derivatives, as described by the group of Brückner [139].

Brückner and co-workers were able to take advantage of the well-known S_N_Ar reactivity of *meso*-pentafluorophenylporphyrins toward nucleophiles at the *p*-F positions to introduce PEG groups to pyrrole-modified porphyrins (PMPs) at a late stage of the synthetic sequence toward *meso*-arylmetalloporpholactones [138,139,155] (Scheme 48). This allowed the preparation of a variety of PEGylated porpholactone metal complexes (**163M**) that were used as optical high pH or cyanide sensors in water [139]. The PEGylated derivatives were purified by silica gel chromatography (CH_2_Cl_2_/5% MeOH). Similarly, thiol-functionalized sugars were introduced into *meso*-pentafluorophenylporphyrins using the S_N_Ar reaction, also resulting in their solubilization in aqueous solutions [156].

**Figure 10:**
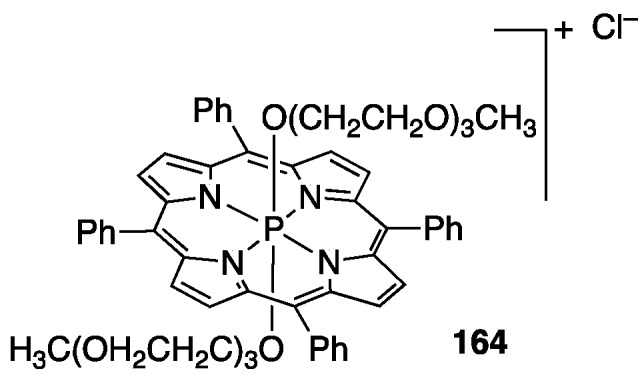
Molecular structure of axially-PEGylated phosphorus porphyrin **164**.

Building on previous efforts for the solubilization of antimony porphyrins by axial ligand exchange with alcohols (cf. Scheme 11), Yasuda and co-workers prepared hydrophilic PEGylated phosphorus porphyrins, such as tetraphenylporphyrin derivative **164** (Figure 10) by introduction of short PEGs as axial ligands. Incorporation of the axial-PEGs furnished highly water-soluble derivatives. Porphyrin **164**, for example, has a water-solubility of 17.3 mM [90].

#### 4.1.3. Porphyrins PEGylated at Their β-Positions

**Scheme 49:**
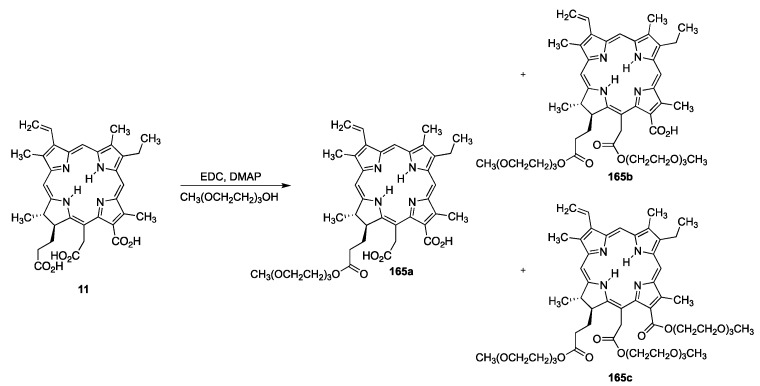
PEGylated derivatives of chlorin e6, as described by the group of Greer [158]. EDC = 1-ethyl3-(3-dimethylaminopropyl)carbodiimide, DMAP = 4-dimethylaminopyridine.

A number of PEGylated β-alkyl-porphyrins and -chlorins were prepared. Representative of these are a number of PEGylated chlorin e6 derivatives, prepared firstly by Hamblin et al. [157]. The PEGylation increased tumor targeting of the photosensitizer. Mono-(**165a**), di-(**165b**), and tri-(**165c**) PEGylated derivatives of chlorin e6 were also prepared in which the PEG groups were introduced by esterification at one to three of its carboxylic acids using a short methyl-capped PEG (Scheme 49) [158]. Longer reaction times were needed to increase the yield of the tri-PEGylated product **165c**. The authors also measured the solubility of the compounds to be 1.8 ± 1.3 mM for **11**, 2.3 ± 1.0 mM for **165a**, 3.3 ± 0.9 mM for **165b**, and 3.9 ± 0.8 mM for **165c** in 1% (*v*/*v*) DMSO/water, indicating the increased aqueous solubility with the increase of the number of PEG chains. Products **165a** and **165b** are single isomers, indicating a sterics-controlled regioselectivity of the sequential PEGylation.

**Scheme 50:**
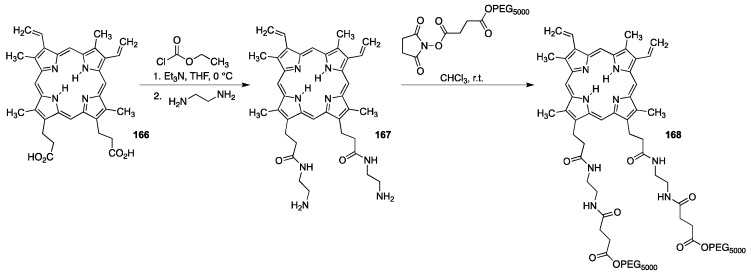
PEGylated protoporphyrin and its zinc complex, as described by the group of Maeda [159].

The propionic side chains in protoporphyrin **166** could also be functionalized with PEG-chains through amide linkages, whereby a short diamine established the requisite amine linkage sites. Amine intermediate **167** was then reacted with succinimidyl-PEG_5000_ to afford the water-soluble PEGylated protoporphyrin derivative **168** (Scheme 50), and its zinc complex [159,160].

**Scheme 51:**
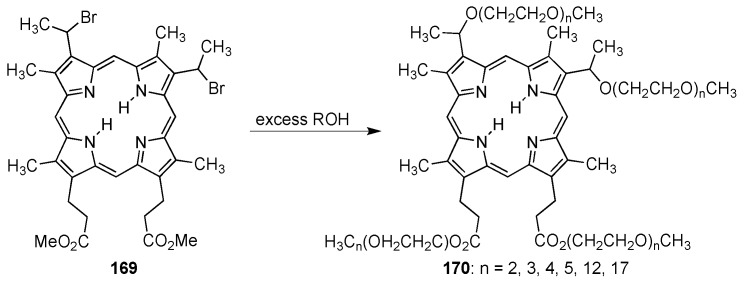
PEGylated hematoporphyrin, as described by Brunner and Sohn [161,162].

Other PEGylated porphyrins were prepared using ether linkages. For example, brominated hematoporphyrin derivative **169** was converted to the corresponding PEGylated compounds **170a**–**170f** by treatment with a stoichiometric excess of a methyl-capped PEG (Scheme 51) [161,162]. Concomitantly, the propionic acid side chains were also derivatized with PEG chains. After hydrolysis of the propionic ester side chains, platinum-conjugates were prepared and evaluated for their antitumor phototoxicty and cytotoxicity [161,162]. The PEGylated derivatives were amenable to aqueous work-up and purified by alumina chromatography.

**Scheme 52:**
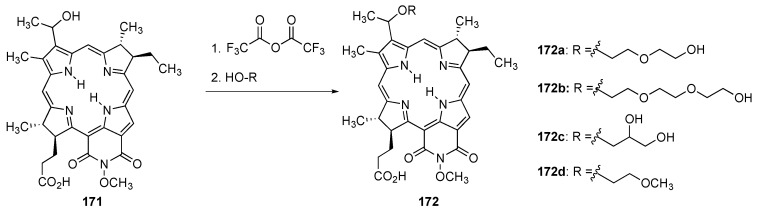
Hydrophilic bacteriochlorin cycloimides, as described by the group of Feofanov [163].

Etherification conditions were also applied to cycloimide-bacteriochlorin alcohol derivative **171** (Scheme 52) [163]. Activation of the hydroxyethyl substituent on the bacteriochlorin **171** with trifluoroacetic anhydride, followed by treatment with hydroxyl-terminated short PEGs and diols of varying length gave the corresponding derivatives **172a**–**172d**. The products were insoluble in water, but aqueous solutions were prepared with the help of 10% Cremophore EL^®^, a PEGylated castor oil formulation vehicle.

**Scheme 53:**
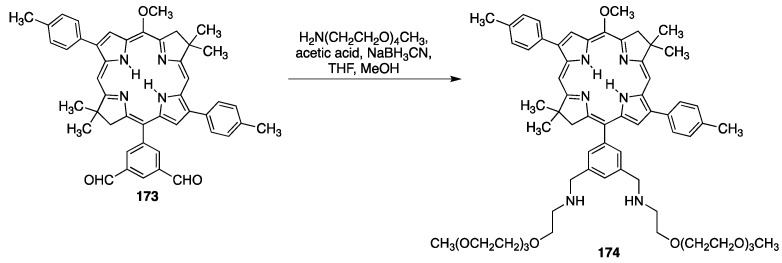
PEGylated bacteriochlorins formed by reductive amination, as described by the group of Lindsey [164].

The *meso*-Suzuki coupling method developed by Lindsey and co-workers and utilized for the introduction of a variety of solubilization groups to hydroporphyrins (cf. Scheme 5, Scheme 12 and Scheme 26) was also utilized for the introduction of PEG groups to bacteriochlorin **173** (Scheme 53) [164]. Bacterio-chlorin **173** was prepared by Suzuki coupling of the corresponding *meso*-bromobacteriochlorin with an aryl-boronic ester bearing two unprotected aldehyde functional groups. The aldehyde functionalities were PEGylated by reductive amination with an amine-terminated PEG to afford the water-soluble PEGylated bacteriochlorin **174**.

**Scheme 54:**
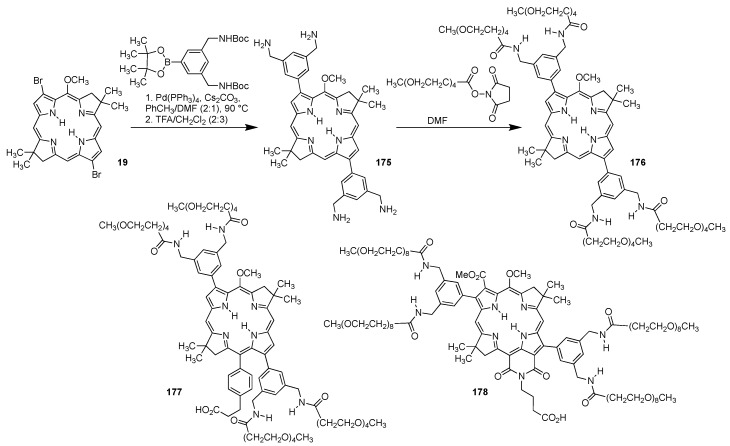
β-Substituted PEGylated synthetic bacteriochlorins via Suzuki coupling, as described by the group of Lindsey [135].

Another representative example for the introduction of PEG groups via Suzuki coupling of dibromobacteriochlorin **19** with a Boc-protected amine-derivatized boronic ester, followed by Boc-deprotection under standard conditions, afforded diaryl bacteriochlorin **175** carrying two amine groups. The amines were PEGylated by amide formation with a PEG-NHS ester to afford bacteriochlorin **176** (Scheme 54) [135]. Derivatives bearing bioconjugatable tethers (**177**) and (**178**) were also prepared using a regioselective *meso*-bromination strategy, followed by similar coupling sequences [135].

**Figure 11:**
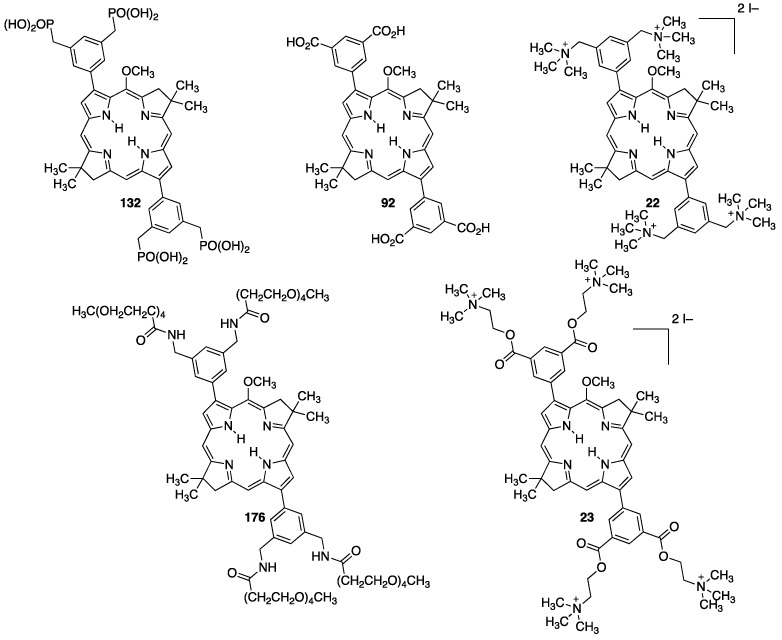
Molecular structures of a variety of examples of water-soluble bacteriochlorins derived by Suzuki coupling (Scheme 54).

Due to the versatility of the 3,13-dibromobacteriochlorin building block **19**, various solubilization motifs could be introduced to this scaffold. Thus, ammonium (compounds **22** and **23**, Figure 11), carboxylate (compound **92**, Figure 11), phosphonate (compound **132**, Figure 11) and PEG (compound **176**, Figure 11) solubilizing motifs were introduced by coupling with suitable arylboronic esters as shown in Scheme 54.

The products were compared by Lindsey and co-workers according to several notable parameters [65] (Table 2). The method of purification of the water-soluble adducts, the solubility of the compounds (as measured by the change in UV-vis absorption spectra over a 1000-fold concentration range), the full width at half-maximum (FWHM) of the longest wavelength of absorption and emission, and the stability of the chromophores in the light and dark.Overall, it was found that either the cationic ammonium or neutral-PEGylated species were the most promising derivatives because of their facile purification (precipitation), their amenability to further bioconjugation reactions, and good dark and light stabilities. The PEGylated derivatives were also shown to display very little aggregation, as demonstrated by the sharp absorbance and emission peaks in their optical spectra over a 1000-fold concentration range.

With the PEGylated derivatives appearing to be among the most promising motifs for solubilization, Lindsey and co-workers set out to prepare a series of PEGylated chlorins and bacteriochlorins (with options for bioconjugation) using a range of synthetic methodologies [135,136]. One such example for the direct, late-stage PEGylation of chlorin derivatives was the azide-alkyne Huisgen-cycloaddition (click) reaction between an alkyne-substituted porphyrin or chlorin and an azide-terminated PEG.

**Scheme 55:**
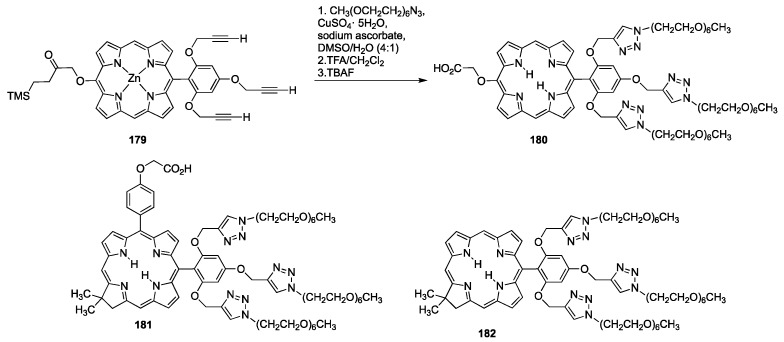
PEGylated porphyrins and chlorins prepared through click chemistry, as described by the group of Lindsey [136].

**Scheme 56:**
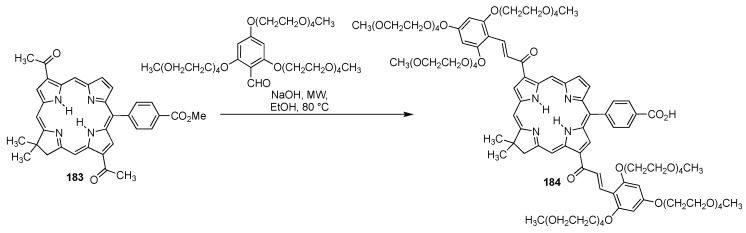
PEGylated chlorins prepared by Aldol-condensation of β-diacetyl chlorin, as described by the group of Lindsey [136].

This strategy was employed to perform a direct PEGylation of *trans*-AB porphyrin **179** (Scheme 55) to afford the PEGylated porphyrin **180** [165]. Along analogous routes, synthetic chlorins **181** and **182** were prepared from alkyne-substituted *meso*-aryl groups and subsequent reactions with PEG-azides [136]. In addition, an aldol condensation method was developed to directly introduce an aryl group bearing three PEG chains to the β-positions of a β-diacetyl-substituted chlorin **183**. Reaction of **183** with a PEG-substituted aryl aldehyde formed the porphyrin-chalcone **184** (Scheme 56) [136]. The absorption spectrum of the PEGylated chlorin-chalcone **184** was broadened in pure water, indicating its aggregation.

**Scheme 57:**
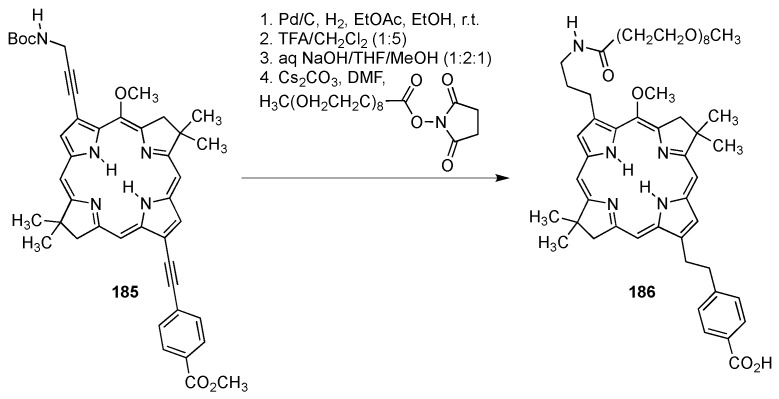
PEGylated bacteriochlorins bearing a bioconjugatable tether prepared by amide coupling, as described by the group of Lindsey [135].

Lindsey and co-workers also used an amide coupling of an alkyl-amine substituted bacteriochlorin with a PEG-NHS ester to produce PEGylated bacteriochlorin **186** (Scheme 57) [135]. The β-functionalized bacteriochlorin **185** was prepared by Sonogashira coupling and subsequent reduction of the alkynes. Boc-deprotection was followed by saponificiation to furnish the amine and acid-derivatized intermediate, which was reacted with a PEG-NHS ester to provide the PEGylated bacteriochlorin **186**. The carboxylic acid functionality on this chromophore is available for bioconjugation.

## 5. Summary and Outlook

In summary, significant progress has been made in the past decades toward the preparation of water-soluble porphyrins and hydroporphyrins. A wide range of options are available for their solubilization in aqueous media. Cationic, anionic or neutral groups may be introduced in regio- and chemoselective fashion. Their number and positioning has major influences on the solubility of the derivatized porphyrin, whereby swallowtail approaches stand out as seemingly particularly effective in mediating water-solubility with a moderate concomitant increase in molecular weight. All solubilizing functionalities can be introduced at early stages of the synthesis of the target compound—generally in protected form—or at late stages. The late-state introduction of the solubilizing motif(s) reduces the number of difficult chromatographic steps needed in the synthetic sequence; and these polar moieties and/or their water-solubility generally complicate their isolation and purification. Irrespective of the timing of the introduction of the solubilizing groups, multiple linking strategies are available. As far as flexibility and ease of implementation, PEGylation strategies stand out because they generate neutral chromophores of good solubility, the PEG chains are chemically stable, the versatility of the linking moieties that can be used to link PEG chains to the chromophore, and the facility of the work-up conditions once the PEG chains are established. A combined approach using both ionic and PEG groups may also have its merits in cases where the introduction of short PEGs was not enough to solubilize the macrocycle.

Even though the toolbox for the synthetic chemist to affect water-solubility is already extensive, the continued development of solubilizing strategies that are mild, selective, and introduce chemically robust solubilization motifs to porphyrins is vital to the field for these versatile chromophores to realize their full potential in the many technical and biomedical applications that take place in aqueous media.

## Figures and Tables

**Table 1:** Comparison of the retention times of hydrophilic porphyrin dimers shown in Scheme 37 [70].

Porphyrin Dimer	Retention Time/min ^a^
51	6.2
29	6.8
135	7.6
136	15.6
88	18.0

^a^ HPLC Conditions: C_8_ column (5 μm, 3.9 mm × 150 mm), 1 mL/min flow rate; solvent A: 1% aqueous acetic acid; solvent B: THF, linear gradient.

**Table 2:** Comparison of relevant properties concerning hydrophilicity of a series of structurally similar synthetic bacteriochlorins, as described by the group of Lindsey [65].

Compounds	Purification Method ^a^	Water-Solubility ^b^	FWHM (abs, fl) (nm) ^c^	Stability in Light (%) ^d^	Stability in Dark (%) ^e^
132	reverse phase chromatography	0.46 μM–460 μM	26, 30	85	>95
92	ppt, washing with hexanes/MeOH (49:1)	0.32 μM–320 μM	26, 26	96	>95
22	ppt, washing with Et_2_O/THF (1:1)	0.24 μM–240 μM	26, 26	82	>95
23	ppt.,washing with Et_2_O/THF (1:1)	0.18 μM–180 μM	40, 26	96	>95
176	ppt, washing with hexanes/CH_2_Cl_2_ (19:1)	0.43 μM–430 μM	22, 23	95	>95

^a^ For the final step of the synthesis; ^b^ measured in aqueous media (PBs except **176**, water) by the absorption upon reciprocal change in concentration and path length; ^c^ 1 μM in PBS. FWHM = full width at half maximum of the longest wavelength band of absorption or emission, an indicator of aggregation; ^d^ The % remaining of a 1 μM solution in PBS after several hours of absorption and fluorescence studies. ^e^ The % remaining of a, 1 μM solution in PBS allowed to stand in the dark at 4 °C for 48 h (as determined by absorption intensity of Q_y_ band.
